# Revealing the Mechanism of Alcohol Side Product Formation in Crown Ether-Mediated Nucleophilic Fluorination Using Acetonitrile as Solvent

**DOI:** 10.1021/acsomega.5c04699

**Published:** 2025-07-16

**Authors:** Eloah P. Ávila, Mauro V. de Almeida, Josefredo R. Pliego

**Affiliations:** † Departamento de Ciências Naturais, 74383 Universidade Federal de São João del-Rei, São João del-Rei, MG 36301-160, Brazil; ‡ Departamento de Química, 28113 Universidade Federal de Juiz de Fora, Campus Universitário, Martelos, Juiz de Fora, MG 36036-330, Brazil

## Abstract

Nucleophilic fluorination of primary alkyl halides using KF salt and catalyzed and mediated by crown ether and bulky alcohols is an established method for monofluorination of organic compounds. However, in the presence of a small concentration of water molecules in the organic solvent, alcohol side products are formed. This is an intriguing finding because water molecules are unreactive toward the S_N_2 reaction. Further, the formation of a hydroxide ion via deprotonation of water by the fluoride ion faces the problem of very different p*K*_a_ values in acetonitrile solution, which were calculated to be 19.5 for HF and 41.9 for H_2_O. This work explores the mechanism behind this side reaction via theoretical calculations and experiments. We found that the deprotonation of H_2_O is driven by the formation of the stable HF_2_^–^ ion, leading to the small concentration of the KOH­(18-crown-6) complex. This species exhibits higher reactivity compared to the KF­(18-crown-6) complex in the S_N_2 process, which offsets its lower concentration and results in a competitive side reaction. Thus, the present study elucidates the mechanism involved in the alcohol side product formation and indicates that the presence of the KHF_2_(18-crown-6) complex can inhibit this side reaction. Furthermore, this work indicates that complex reaction systems require an analysis beyond the comparative barriers in the free energy profile, and multiple equilibria must be accounted for.

## Graphical abstract

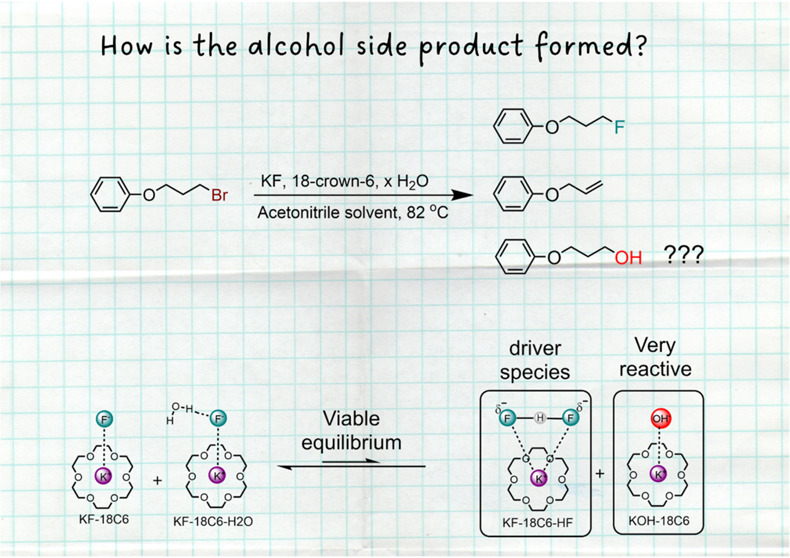

## 1 Introduction

Fluorination reactions of aliphatic substrates have received increased interest in the past two decades.^1–5^ Many interesting new pharmaceuticals with C­(sp^3^)–F bond have been approved in recent years.^6,7^ Additionally, useful applications in materials chemistry have been reported,^8^ inducing more developments in fluorination reactions. As examples, [^18^F]­Cyclofoxy and [^18^F]­fluoroethylflumazenil are radiopharmaceuticals used in the diagnostic imaging of neurological diseases (Figure 1). The improvement of the anti-inflammatory activity of ibuprofen was observed when a methyl hydrogen atom was replaced by a CH_2_F group, which was three times more potent than its nonfluorinated (*S*)-ibuprofen analogue against COX-1.^9–11^ In the field of materials chemistry, incorporating a fluorine atom can be utilized to modify the optical properties of π-conjugate systems within chlorophyll motifs, thus enhancing their application in photosynthetic antenna.^12^

**Figure 1:**
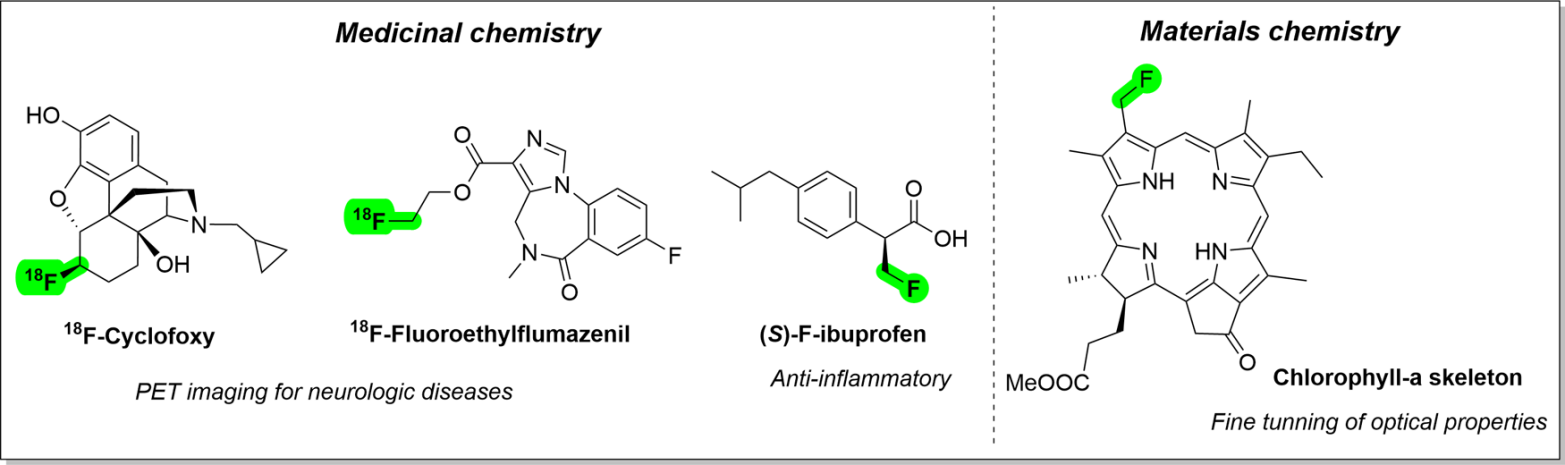
Applications of the Alkyl fluorine motifs.

Among different reactants, nucleophilic fluorination using KF salts as a fluorine source is very interesting from a green chemistry perspective.^13–15^ However, these reactions face important challenges such as solubilization of KF in organic solvents and high solvation of the fluoride ion in polar protic solvents, resulting in low reactivity.^16^ A pathway for overcoming these problems is the use of crown ether and derivatives, and control of hydrogen bonds, creating structured environments able to solubilize and activate the fluoride salts, producing selective fluorination.^17–30^ Recently, our group has investigated a combination of crown ether with diverse fluorinated bulky alcohols^31^ as a more effective fluorination method than the use of crown ether alone, or crown ether combined with *tert*-butyl alcohol. The method has worked very well for a primary alkyl bromide substrate with 89% conversion in just 6h of reaction at a mild temperature of 82 °C when using 2 equiv of 18-crown-6 (18C6) and 6 equiv of 1,1,1-trifluoro-2-methyl-2-propanol (TBOH-F3) in acetonitrile solvent. It was reported that there was formation of 70% of the fluorinated product, 5% of the E2 product (S_N_2:E2 ratio of 93:7), and 14% of the corresponding alcohol. This alcohol was formed from the reaction of the primary alkyl bromide substrate with water present in the medium. Although the solvents and reactants could be dried to decrease the concentration of water, resulting in less alcohol product, it is not possible to eliminate water completely in real reaction conditions.^32^ Aimed at improving selective fluorination, it is essential to understand the mechanism of alcohol formation to develop new methods that completely suppress this side product.

The fluorination experiments^31^ have also used KF and 18-crown-6 without fluorinated bulky alcohol, and the results are presented in Scheme 1. This process leads to a substantial alcohol yield (13%), and although this side reaction is a shortcoming, the water molecules present in the medium also induce more S_N_2:E2 selectivity. Hence, the water present in the medium has both an advantage, a higher S_N_2:E2 product ratio, and a disadvantage, the formation of an alcohol side product. A related finding is the formation of an ether side product when hexafluorinated *tert*-butyl alcohol (TBOH-F6, p*K*_a_ = 26.2 in acetonitrile) is used, and the absence of an ether product when less acidic trifluorinated *tert*-butyl alcohol (TBOH-F3, p*K*_a_ = 35.0 in acetonitrile) is used. For comparison, the p*K*_a_ of water in acetonitrile was calculated to be 41.9 units.^31^ These results appear contradictory and warrant further investigation.

**Scheme 1:**
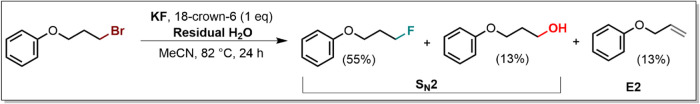
Formation of Alcohol Side Product in the Crown Ether Mediated Fluorination Using Non-Dried Acetonitrile Solvent^a^

a Yields taken from ref 31. Products determined by ^1^H NMR of crude reaction, 0.25 M of substrate.

Understanding the real mechanism behind alcohol (and ether) formation in this kind of system is a fundamental problem in chemistry. Thus, in this work, we have performed a profound computational exploration of the effect of water molecules and the possible reaction pathways for the formation of alcohols from water present in the medium. The possible mechanisms are presented in Scheme 2. The first and most obvious possibility is the direct reaction of water molecules with the primary alkyl bromide substrate. A second possibility is the formation of the most reactive hydroxide ion, which forms an ion pair with the potassium ion coordinated by 18-crown-6. This KOH­(18C6) species could be generated by decomposition of the KF­(18C6) complex coordinated by one water molecule, eliminating HF and forming KOH­(18C6). This released HF could encounter another KF­(18C6) complex, forming the HF_2_^–^ ion^33^ coordinated to the potassium cation in 18-crown-6 (Scheme 1). This equilibrium, presented in Scheme 2, and the kinetics of the reaction of KOH­(18C6) with the primary alkyl bromide substrate are critical to determine the competition between the formation of alcohol and the alkyl fluoride products. These mechanisms were explored in this work for the reaction in Scheme 1.

**Scheme 2:**
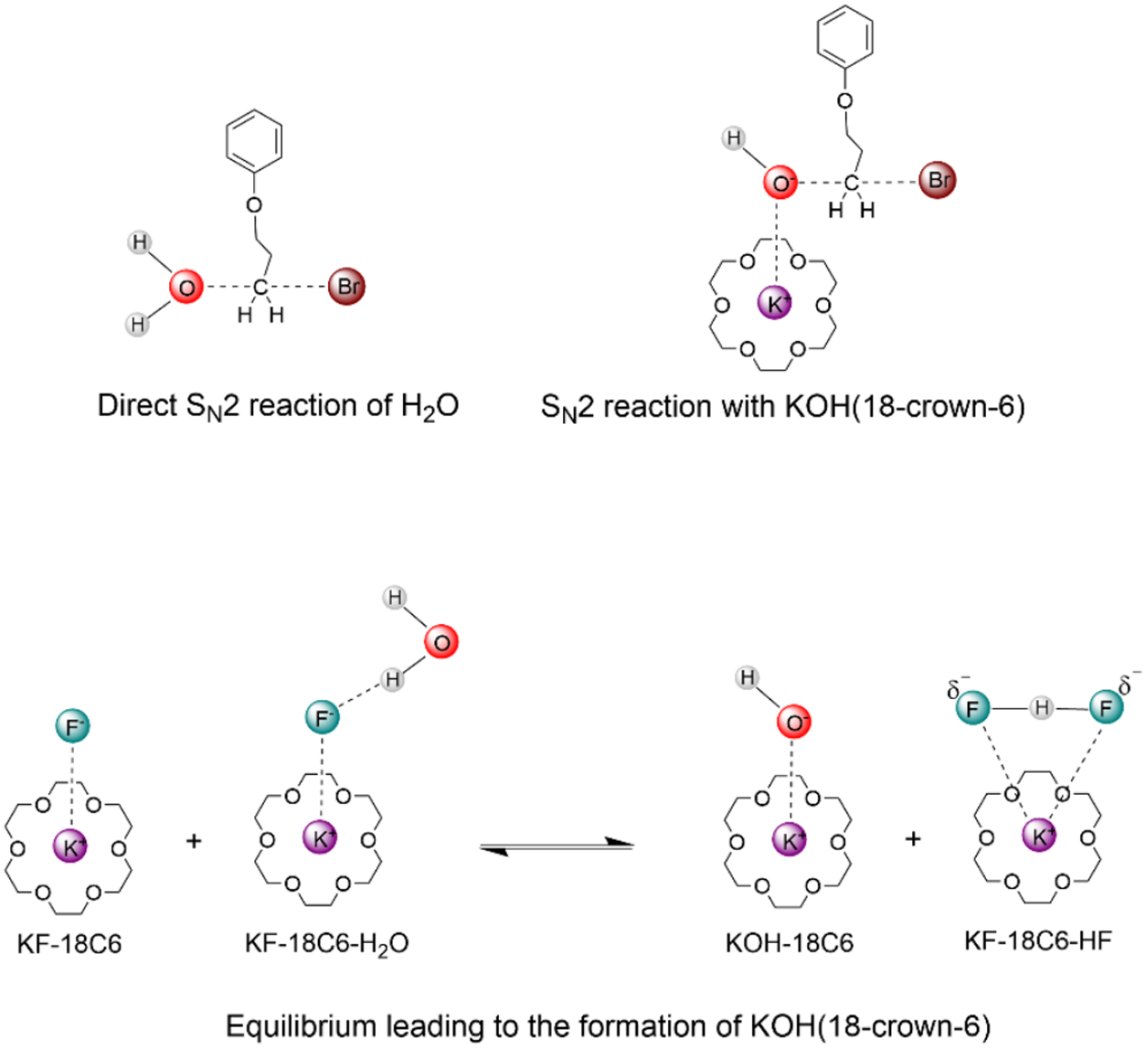
Possible Mechanisms for the Formation of the Alcohol Side Product

## 2 Results and Discussion

### 2.1 Direct Reaction with Water

The first mechanistic possibility for alcohol formation is the direct S_N_2 reaction of water present in the medium with the primary alkyl bromide substrate. The located transition state and the respective free energy barrier are listed in Figure 2. As we can see, the free energy barrier is very high, reaching 40.6 kcal mol^–1^, and leading to very slow kinetics. Consequently, this reaction does not take place under experimental conditions (82 °C), indicating that another mechanism plays the role.

**Figure 2:**
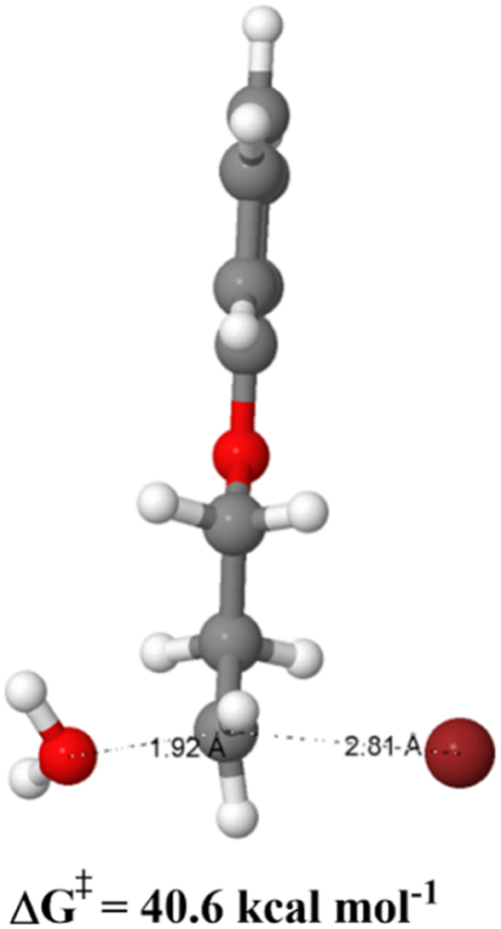
Transition state for direct water reaction with the primary alkyl bromide substrate. Calculations at the CPCM/ωB97M-V/ma-def2-TZVPP//CPCM/X3LYP/ma-def2-SVP level of theory are made using acetonitrile solvent.

### 2.2 Formation of Hydroxide Ion

Once the water molecule is unreactive toward the S_N_2 process, the more reactive hydroxide ion must be the active species. The problem is the unfavorable thermodynamics for the formation of the OH^–^ ion because HF is much more acidic than H_2_O in acetonitrile solution. Thus, considering their p*K*_a_ data,^31^ we can write

1 $$H_{2}O+F^{-}\to {\mathrm{OH}}^{-}+\mathrm{HF},\;\Delta G=30.6{\mathrm{kcal mol}}^{-1}$$

However, this free energy data is related to the free solvated species in acetonitrile, while the small fluoride and hydroxide ions are forming ion pairs with K^+^(18-crown-6) in acetonitrile solution.^34,35^ Thus, the role of countercation and the crown ether should be considered to elucidate the mechanism of formation of the hydroxide ion. The related calculations are presented in Scheme 3. We can see that when one crown ether and one water molecule are considered, the free energy for the formation of the KOH­(18C6) complex from the initial reactants (solid KF) is 32.1 kcal mol^–1^, making this process inviable. Even the addition of a second water molecule is not able to make the process viable because the free energy becomes 27.3 kcal mol^–1^ with the formation of the KOH-18C6-H_2_O complex. Thus, the interactions with the crown ether and the counterion may not account for this reaction.

**Scheme 3:**
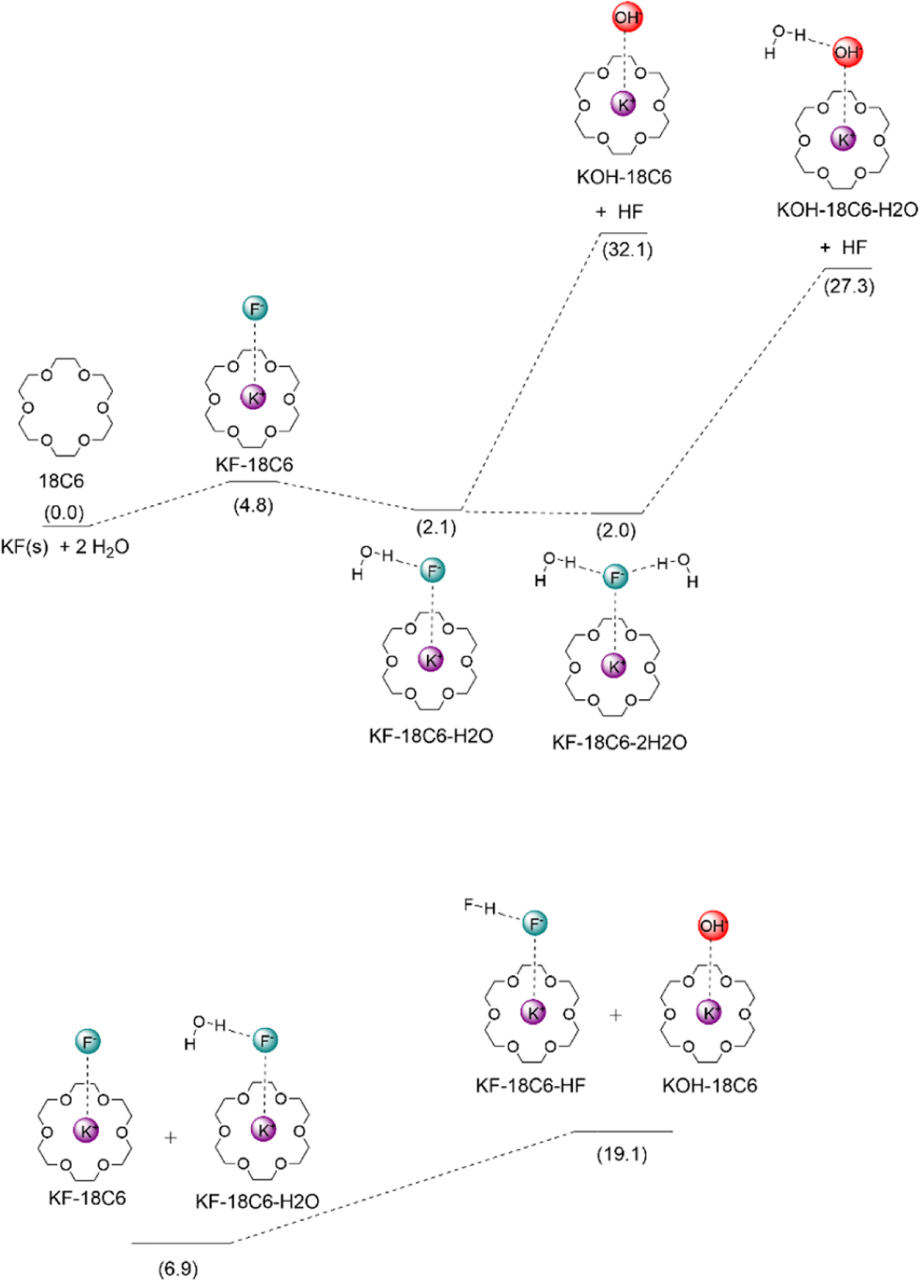
Possible Mechanisms Leading to the Formation of the Hydroxide Ion^a^

a Units in kcal mol^–1^, 25 °C, standard state of 1 mol L^–1^.

Another possibility of lowering the high free energy barrier for the deprotonation of H_2_O is the formation of stable HF_2_^–^ species. This possibility is presented in Scheme 3, which exhibits the initially formed complexes KF-18C6 and KF-18C6-H_2_O, along with their free energies. This acid–base reaction leads to the formation of the KF-18C6-HF and KOH-18C6 complexes with a reduced overall free energy cost of 19.1 kcal mol^–1^. This substantial stabilization can be enough for promoting alcohol formation via the reaction of the KOH-18C6 complex with the primary alkyl bromide substrate. Because the hydroxide ion is a very reactive species in the gas phase,^36,37^ and considerably less reactive in aqueous solution,^38^ this less solvated environment (acetonitrile) could lead to very high S_N_2 reactivity of the soluble KOH-18C6 species. The competition between these side reactions depends on the relative barriers for the reaction of KF-18C6 and KOH-18C6 with the substrate. Considering that multiple equilibria and several parallel pathways take place, a detailed kinetics analysis is needed, which is done in a discussion in this text. As a first step, we need to calculate the reaction free energy profiles.

### 2.3 Effect of Water Molecules on the Fluorination Reaction

The free energy profile for the KF­(18C6) reaction with the primary alkyl bromide substrate, including the effect of water molecules present in the medium, is shown in Scheme 4. When the solubilization equilibrium is included (4.8 kcal mol^–1^), the free energy barriers for the reaction via S_N_2 (TS1-18C6) and E2 (T2-18C6) mechanisms involving only the crown ether are 25.9 and 26.0 kcal mol^–1^, respectively, indicating that almost 50% of the E2 product should be formed. The water molecules present in the medium can form a complex with the KF­(18C6) species, with Δ*G* = −2.8 kcal mol^–1^, increasing the solubilization of KF. Thus, the KF-18C6-H_2_O species has a free energy of 2.0 kcal mol^–1^ in the diagram. This complex can also react through S_N_2 and E2 pathways via the TS1-18C6-H_2_O and TS2-18C6-H_2_O transition states. Their overall free energy barriers in the diagram are 26.4 and 30.8 kcal mol^–1^, respectively. Thus, the water molecules in the S_N_2 and E2 transition states can produce a very different stabilization of these pathways and lead to a very favorable S_N_2 mechanism. However, because multiple equilibria take place with different concentrations of KF-18C6 and KF-18C6-H_2_O species, the S_N_2:E2 selectivity should be highly dependent on the water concentration. An increase in the water concentration is expected to result in a decrease in the E2 yield, which aligns with experimental observations (Section 2.5). Indeed, the reaction with lower water concentration (not dried solvent) resulted in an 81:19 product ratio (S_N_2:E2), while the use of 0.75 M water resulted in 92:8 selectivity favored for S_N_2 (Section 2.5). A kinetic analysis is done in a posterior section.

**Scheme 4:**
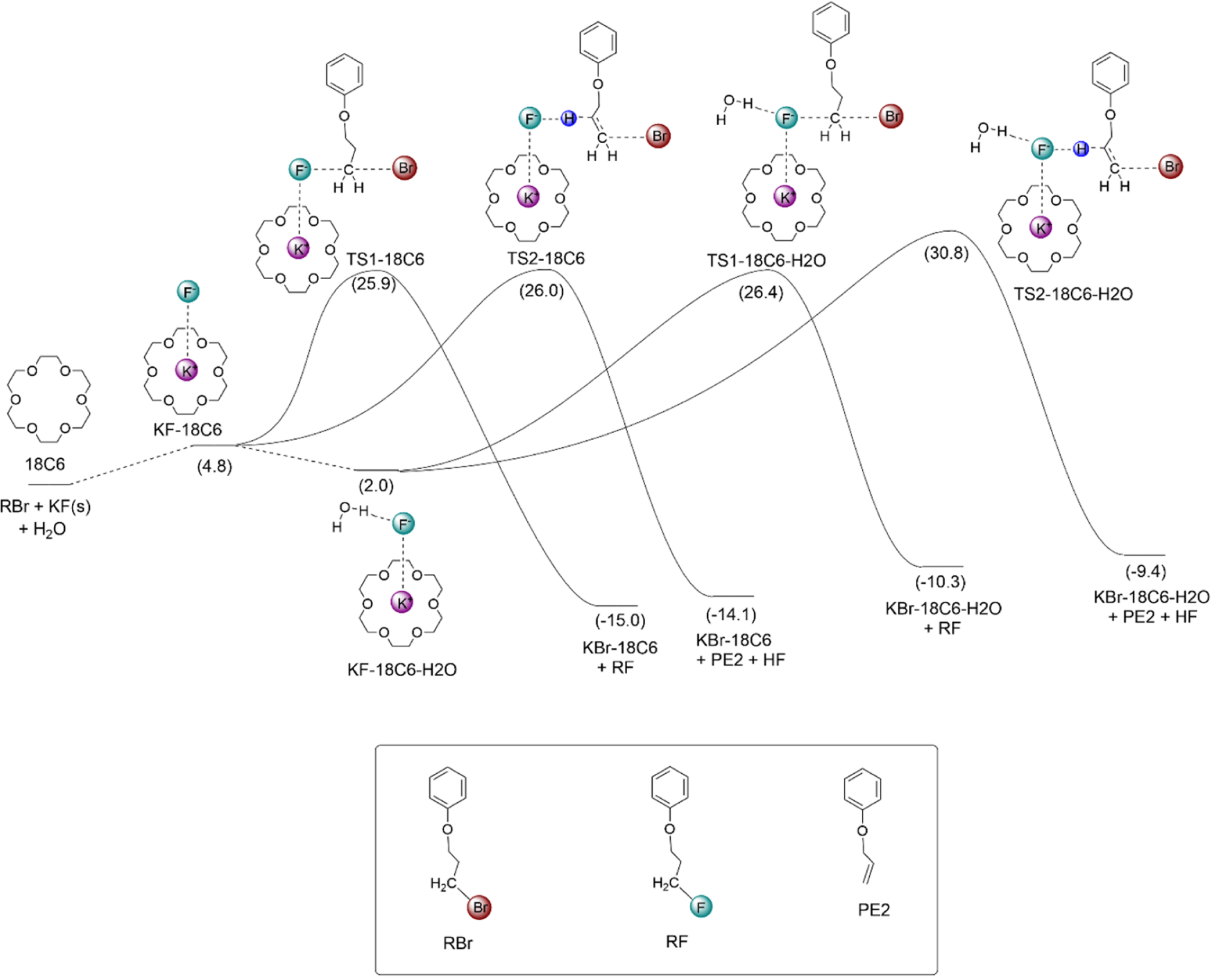
Free Energy Profile for the Fluorination Reaction^a^

a Units in kcal mol^–1^, 25 °C, standard state of 1 mol L^–1^.

### 2.4 Free Energy Profile for the Alcohol Formation

Aimed at analyzing the viability of the alcohol product to be formed from the KOH-18C6 species, we have investigated its reactivity with the primary alkyl bromide substrate. The free energy profile is presented in Scheme 5. The S_N_2 reaction via TS3-18C6 leads to the formation of the alcohol product, while the reaction via TS4-18C6 is the E2 product. Both barriers are 14.9 kcal mol^–1^, indicating a high competition between these pathways. For comparison, the Δ*G* ^‡^ for fluorination from the KF-18C6 complex is 21.1 kcal mol^–1^, showing that the hydroxide ion bound to the K^+^(18C6) complex is much more reactive than the fluoride ion in this complex, with ΔΔ*G* ^‡^ = 6.2 kcal mol^–1^. This very high reactivity can compensate for the very low concentration of KOH-18C6 species in the solution phase under the fluorination reaction conditions.

**Scheme 5:**
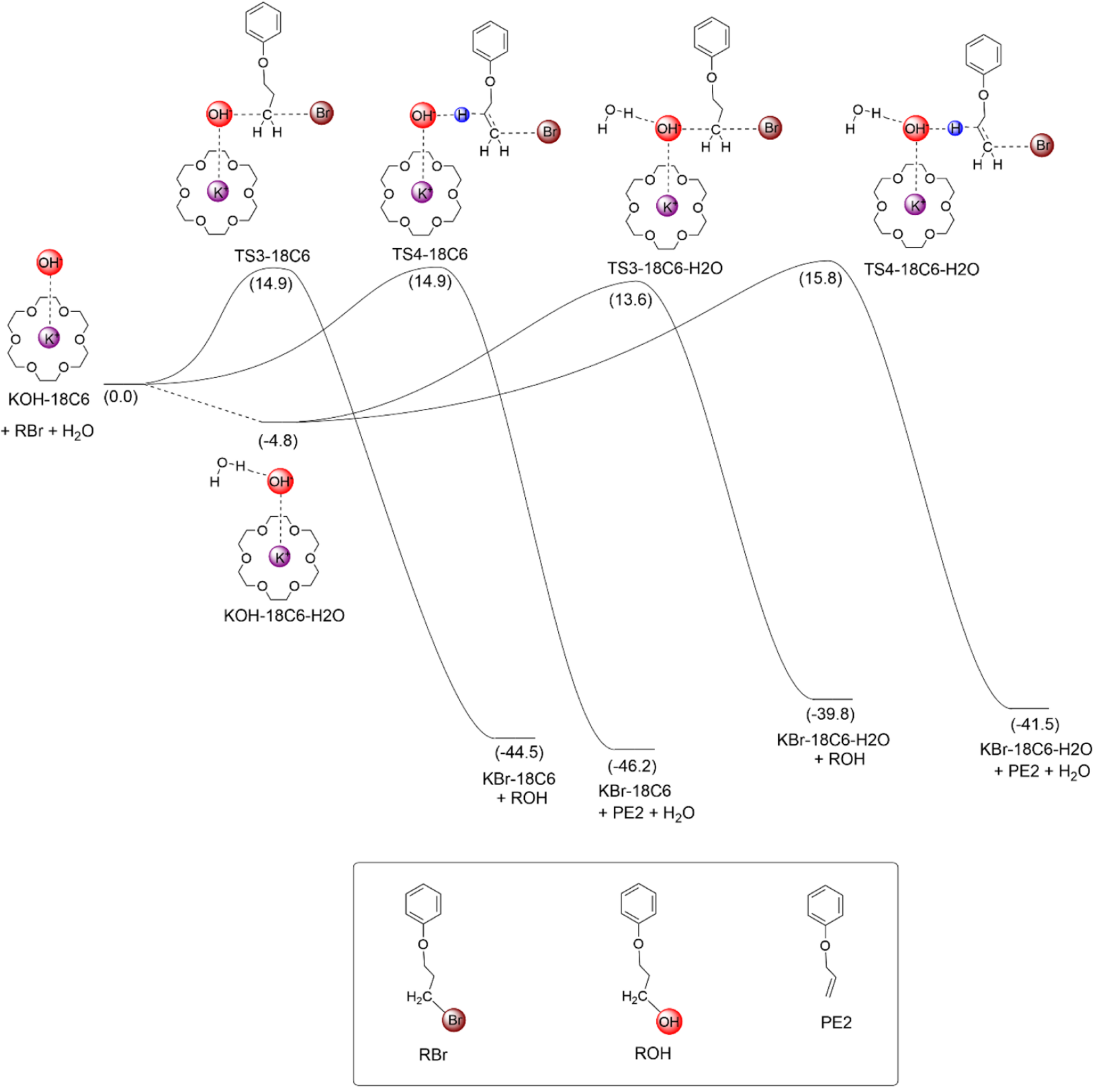
Free Energy Profile for the Alcohol Formation Reaction, Considering the KOH-18C6 Complex as the Reference Species^a^

a Units in kcal mol^–1^, 25 °C, standard state of 1 mol L^–1^. The free energy for the process KOH(s) + 18C6 → KOH­(18C6) was calculated as 0.54 kcal mol^–1^, data not included in the diagram.

Because water molecules are present in the medium, we also analyzed the effect of the water molecules on the free energy profile. The addition of one water to the KOH-18C6 complex decreases the free energy by 4.8 kcal mol^–1^, indicating a high stabilization of the KOH-18C6-H_2_O complex. The respective free energies of the S_N_2 (TS3-18C6-H_2_O) and E2 (TS4-18C6-H_2_O) transition states are 13.6 and 15.8 kcal mol^–1^, respectively. The Δ*G* ^‡^ barriers from the KOH-18C6-H_2_O complex become 18.4 and 20.6 kcal mol^–1^, respectively. Consequently, the water present in the medium has a very important effect on the selectivity. First, the E2-S_N_2 difference in the Δ*G* ^‡^ barriers becomes 2 kcal mol^–1^, reducing E2 via these transition states, and second, because the S_N_2 pathway via TS3-18C6-H_2_O is 1.3 kcal mol^–1^ below the E2 pathway via TS4-18C6. Thus, the alcohol product must be the major product when KOH-18C6-H_2_O is the reactant, in agreement with the experiments at the beginning of the reaction (Section 2.6). In summary, the KOH-18C6 complex is very reactive and selective toward alcohol formation in the presence of water. Thus, the mechanisms in Schemes 3 and 5 could explain the alcohol side product formation in nucleophilic fluorination with KF mediated by crown ether. A detailed kinetic analysis to better understand these multiple equilibria and reaction pathways is provided in the following sections.

### 2.5 Experiments on the Effects of Water Molecules on the Reaction of the KF­(18C6) with the Primary Alkyl Bromide

The effect of water on the fluorination reaction is presented in Figure 3. When only water contaminant is present (from not dried solvent), we can see that the kinetics of total conversion depend on the crown ether concentration, with 26% conversion using 0.5 equiv of 18C6 at 4 h of reaction, and 37% conversion using 1.0 equiv of 18C6 in the same reaction time. In addition, substantial E2 product is generated (S_N_2:E2 selectivity around 80:20), and the alcohol product is also formed in an appreciable amount. The addition of more water molecules to the reaction (up to 0.75 M) hardly affects the kinetics in the experiments containing 1 equiv of 18C6, with both cases reaching 80% conversion after 24(26) hours. This finding is in line with Scheme 4, where the barriers via TS1-18C6 and TS1-18C6-H_2_O are close. On the other hand, the addition of more water molecules suppressed the E2 product, with S_N_2:E2 selectivity improving to 92:8. This observation is also qualitatively in line with Scheme 4, because the pathway via TS2-18C6-H_2_O is less favorable. Finally, the yield of the alcohol product slightly increased at 24 h of reaction from 13% to 15%, and the fluorination yield also increased from 55% to 60%. Therefore, the addition of water has some advantages such as E2 suppression and a consequent increase in chemoselectivity.

**Figure 3:**
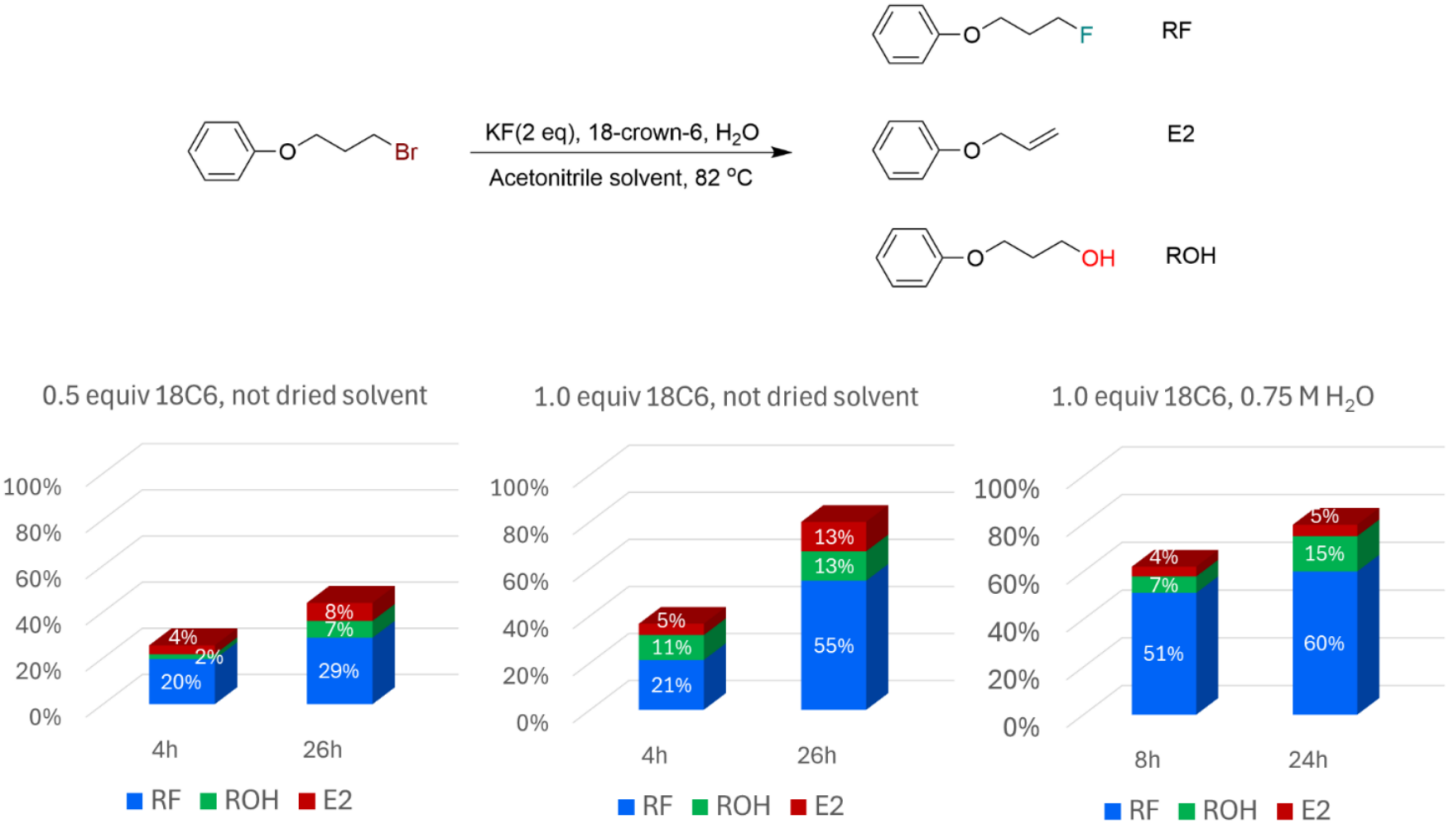
Effect of water on the fluorination of a primary alkyl bromide with KF catalyzed and mediated by 18-crown-6 in acetonitrile solution. Substrate at 0.25 M. Yields at different times. Data for reactions at 24 h (26 h) taken from ref 31

A simple kinetics analysis can be done to estimate the experimental barrier via TS1-18C6-H_2_O. Thus, considering the solubilization equilibrium to form KF­(18C6)­(H_2_O) and its reaction, the kinetic law becomes

2 $$\frac{d\left[\mathrm{RBr}\right]}{dt}=-k{\left[18C6\right]}_{0}{\left[H_{2}O\right]}_{0}\left[\mathrm{RBr}\right]$$

With pseudo-first-order kinetics given by

3 $$\left[\mathrm{RBr}\right]={\left[\mathrm{RBr}\right]}_{0}e^{-k{\left[18C6\right]}_{0}{\left[H_{2}O\right]}_{0}t}$$

Considering 8h of reaction and 62% conversion and that [H_2_O] = 0.75 M, [18C6] = 0.25 M, the rate constant is calculated to be k = 1.8 × 10^–4^ M^–2^ s^–1^. Using transition state theory, we can calculate that at 82 °C, Δ*G* ^‡^ = 27.0 kcal mol^–1^. This estimated experimental value is in excellent agreement with the theoretically calculated Δ*G* ^‡^ = 26.4 kcal mol^–1^ from Scheme 4.

### 2.6 Experiments on the Reaction of KOH­(18C6) with the Primary Alkyl Bromide and the Effect of Water Molecules

In this part of the study, our goal was to evaluate the direct reaction of H_2_O and OH^–^ mediated by crown ether, with the primary alkyl bromide substrate. Initially, we evaluated the ability of neutral water as the nucleophile, using 0.75 M of water dissolved in acetonitrile. After 24 h at 82 °C, the reaction product was almost indetectable (see SI), in line with the calculations, which indicated very slow kinetics. In the sequence, we investigated the reaction of KOH mediated with crown ether in acetonitrile (not dried) and also this same reaction in the presence of 0.75 M of water. Both reactions were monitored for a period of 6h at a lower temperature of 50 °C. The results are in Figure 4. We can observe that the reaction proceeds quickly, with more than 70% conversion in just 2h of reaction for both the cases. In this time, the alcohol product is the major one, mainly in the case of the use of 0.75 M of H_2_O. This finding is in line with Scheme 4, indicating that water molecules induce more selectivity, favoring S_N_2. Furthermore, even at this time, it is possible to see the formation of the ether product (ROR, dimer of the alcohol). This species can be formed from the deprotonation of the alcohol and its subsequent S_N_2 reaction with the substrate. As the reaction advances, the yield of the alcohol product drops slightly, and we see an increase in the E2 product. It seems that deprotonated alcohol, formed in the medium, works better as a base than hydroxide ion and induces more E2 product. In the latest reaction time (6h), we observed conversions of 85% (not dried acetonitrile) and 95% (0.75 M of water). In addition, it is evident that the reaction with KOH is substantially faster than with KF because we observed a higher conversion in a lower temperature. This observation is also in qualitative agreement with those of Schemes 4 and 5. On the other hand, the reaction using undried acetonitrile and using 0.75 M of water has similar kinetics, although in the last case, the reaction was slightly faster, contrary to Scheme 5. This finding may be explained by the complex kinetics involved, including the rate of KOH dissolution.^39–42^ Thus, it is possible that water molecules could help the kinetics of KOH dissolution, and in the case of less water in the medium, the dissolution kinetics could become rate-determining.^39–42^ Indeed, the barrier computed in Scheme 5 for the KOH­(18C6) reaction is very low, indicating that another important event is limiting the kinetics.

**Figure 4:**
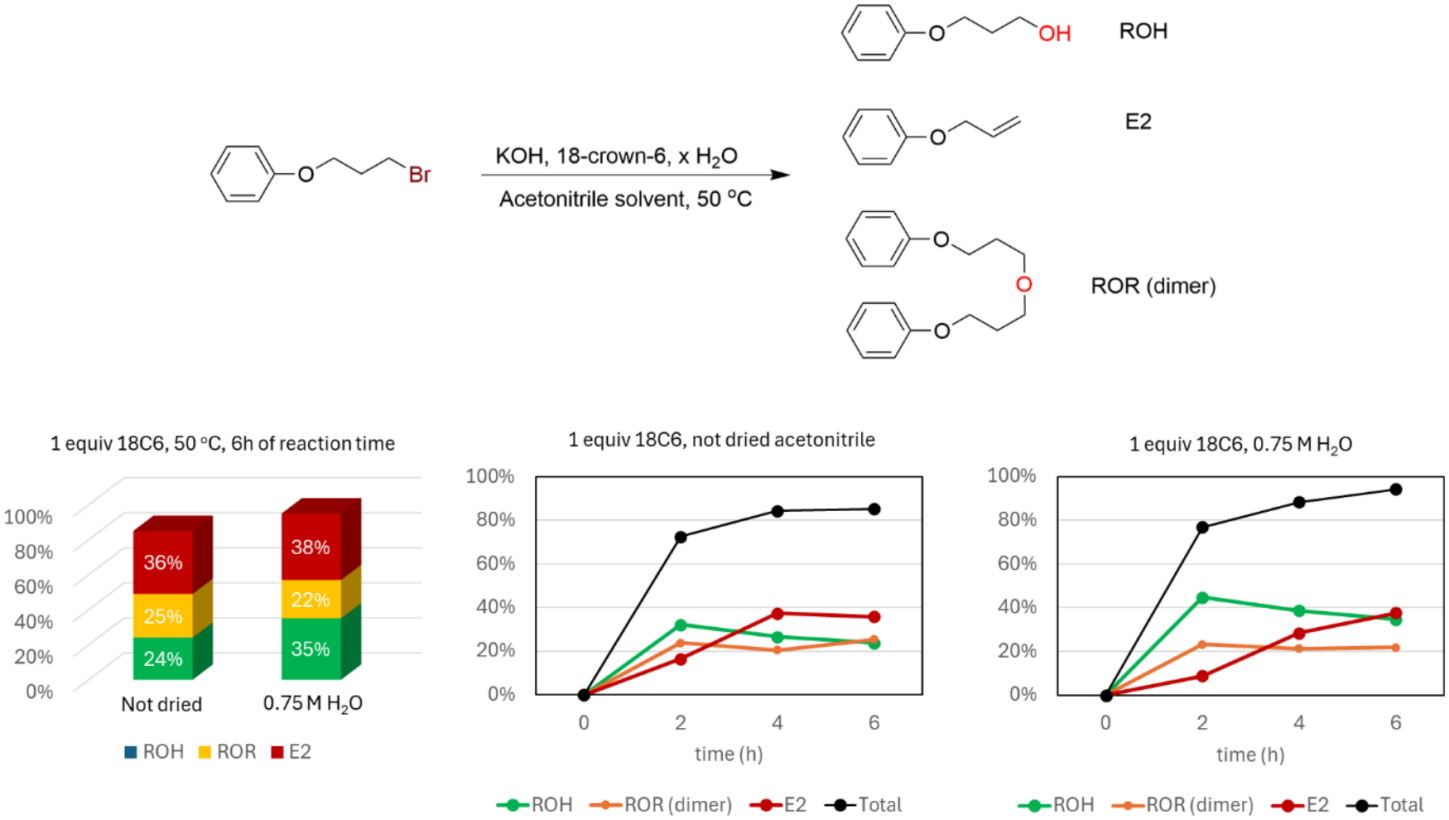
Reactivity of KOH with a primary alkyl bromide mediated by 18-crown-6 in acetonitrile solution and the effect of water. Yield versus time, substrate at 0.25 M, and temperature at 50 °C.

Aimed at comparing our theoretical free energy barrier for the reaction involving KOH-18C6-H_2_O with the experiments (using 0.75 M H_2_O), we can use an approximated kinetic model and the conversion at 2h of reaction time. Thus, based on Scheme 5, considering that the kinetics is given by

4 $$\frac{d\left[\mathrm{RBr}\right]}{dt}=-k\left[\mathrm{KOH}\left(18C6\right)H_{2}O\right]\left[\mathrm{RBr}\right]$$

and the same initial concentrations of these reactants, the kinetic law becomes:

5 $$\frac{1}{\left[\mathrm{RBr}\right]}=\frac{1}{{\left[\mathrm{RBr}\right]}_{0}}+\mathrm{kt}$$

With a conversion of 77% and an initial concentration of 0.25 M, the rate constant is *k* = 1.9 × 10^–3^ M^–1^ s^–1^. Considering the temperature of 50 °C, the transition state theory leads to Δ*G* ^‡^ = 23.0 kcal mol^–1^, compared with the theoretical value of 18.4 kcal mol^–1^. The deviation is relatively high, and a possibility to explain this difference is the formation of a more stable complex, KOH­(18C6)­(H_2_O)_2_, with two water molecules. The calculated variation of the free energy for the KOH­(18C6)­(H_2_O) + H_2_O → KOH­(18C6)­(H_2_O)_2_ process is −2.2 kcal mol^–1^. Thus, considering that this more stable KOH­(18C6)­(H_2_O)_2_ complex needs to dissociate to generate the more reactive KOH­(18C6)­(H_2_O) species, the cost of 2.2 kcal mol^–1^ needs to be added to the barrier of 18.4 kcal mol^–1^, resulting in an effective Δ*G* ^‡^ = 20.6 kcal mol^–1^. This value differs by only 2.4 kcal mol^–1^ from the experimental one. Therefore, our calculations are in good agreement with the experiments regarding the observed kinetics for the reaction with 0.75 M of water.

### 2.7 Theoretical Kinetics Analysis of the Effect of Water on the S_N_2:E2 Selectivity in the Fluorination Reaction

The solubilization of KF by crown ether in an acetonitrile solution is strongly dependent on the water concentration. This claim is supported by both theoretical calculations (Scheme 4) and experimental data.^43^ The reported experimental data indicated that the solubility of KF in dried acetonitrile solution when using 0.11 M concentration of 18C6 is 9.4 × 10^–4^ M at 25 °C.^43^ Considering the solubilization equilibrium:

6 KF(s)+18C6⇌KF‐18C6

The related equation is

7 $$K_{1}=\frac{\left[\mathrm{KF}‐18C6\right]}{\left[18C6\right]}$$

And we can calculate the experimental free energy of solubilization as

8 $${\Delta G}_{1}=-\mathrm{RT}\;\ln\left(K_{1}\right)=-\mathrm{RT}\;\ln\left(\frac{\left[\mathrm{KF}‐18C6\right]}{\left[18C6\right]}\right)=2.8\mathrm{kcal}\;{\mathrm{mol}}^{-1}$$

In this calculation, we have considered all of the soluble KF as complexed with 18C6. Hence, this experimental value can be compared with the theoretically calculated value of 4.8 kcal mol^–1^ (Scheme 3), a very good agreement, with a deviation of only 2.0 kcal mol^–1^.

As noted by Pollard and co-workers,^43^ it is not possible to obtain zero water concentration on experimental conditions. The usual anhydrous conditions used in the laboratory correspond to [H_2_O] close to 3 × 10^–4^ M, while usual, not dried acetonitrile has [H_2_O] close to 8 × 10^–3^ M.^32^ It is worth estimating how the normal concentration of water alters the solubilization and relative concentration of species. Thus, considering the complexation equilibrium for the formation of KF-18C6 at 25 °C:

9 $$\mathrm{KF}‐18C6+H_{2}O⇌\mathrm{KF}‐18C6-H_{2}O$$

10 $$K_{2}=\frac{\left[\mathrm{KF}‐18C6‐H_{2}O\right]}{\left[\mathrm{KF}‐18C6\right]\left[H_{2}O\right]}=113$$

We can estimate that

11 $$\frac{\left[\mathrm{KF}‐18C6‐H_{2}O\right]}{\left[\mathrm{KF}‐18C6\right]}=K_{2}\left[H_{2}O\right]=0.90$$

Therefore, the calculations indicate that not dried acetonitrile is able to double the concentration of solubilized KF. Furthermore, both species are present in the solution phase and can determine the product ratio.

Aimed to determine how the water concentration determines the product ratio, the equilibrium and reaction pathways in Scheme 4 need to be included in a kinetic model.^44^ Thus, considering all the species that have the crown ether and water, we can write

12 $$C_{18C6}=\left[18C6\right]+K_{1}\left[18C6\right]+K_{1}K_{2}\left[18C6\right]\left[H_{2}O\right]$$

13 $$C_{H_{2}O}=\left[H_{2}O\right]+K_{1}K_{2}\left[18C6\right]\left[H_{2}O\right]$$

Resulting that

14 $$\left[18C6\right]=\frac{C_{18C6}}{1+K_{1}+K_{1}K_{2}\left[H_{2}O\right]}$$

15 $$\left[H_{2}O\right]=\frac{C_{\mathrm{ROH}}}{1+K_{1}K_{2}[18C6]}$$

Equations 14 and 15 need to be resolved self-consistently using a spreadsheet for each total (analytic) concentration of crown ether and water. In the sequence, the concentrations of KF-18C6 and KF-18C6-H_2_O can be determined from eqs 7 and 10. With these data and the rate constants via TS1-18C6 and TS2-18C6 from KF-18C6 reference and via TS1-18C6-H_2_O and TS2-18C6-H_2_O from KF-18C6-H_2_O reference (Scheme 4), the reaction rate at the beginning of the reaction and the respective S_N_2:E2 selectivity were calculated. The results are presented in Figure 5. The results show a high E2 product in very low water concentration (54% S_N_2 selectivity). An enhanced increase in S_N_2 selectivity is observed when the water concentration reaches 0.10 M, achieving 61% selectivity at a concentration of 0.75 M. For comparison, in the experiments, 92% selectivity was obtained at this water concentration. The calculations extend to 10 M of water, predicting a selectivity up to 87%. The present results show that the calculations are in reasonable agreement with the experiments, and it is important to consider the uncertainty of the calculated free energy data. In summary, our free energy profile and kinetic model can explain the experiments regarding increasing the S_N_2:E2 selectivity with the increase in water concentration.

**Figure 5:**
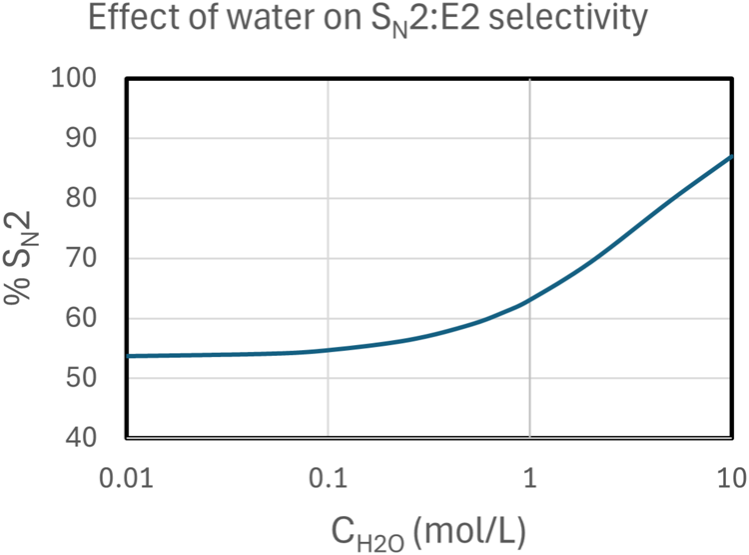
Calculated selectivity of S_N_2 and E2 pathways as a function of the total water concentration, considering eqs 7, 10, 14, and 15 and the free energy profile of Scheme 4, using 0.25 M substrate and 0.25 M crown ether, temperature at 82 °C.

### 2.8 Theoretical Kinetics Analysis of Alcohol Side Product Formation in the Fluorination Reaction

The next step for explaining this reaction system is the inclusion of alcohol formation in the kinetic model, described in Schemes 3 and 5. The first equilibrium to be included is the formation of the KOH-18C6 species, given by

16 $$K_{3}=\frac{\left[\mathrm{KF}‐18C6‐\mathrm{HF}\right]\left[\mathrm{KOH}‐18C6\right]}{\left[\mathrm{KF}‐18C6\right]\left[\mathrm{KF}‐18C6‐H_{2}O\right]}$$

Thus, the concentration of KOH-18C6 species can be written as

17 $$\left[\mathrm{KOH}‐18C6\right]=\frac{K_{3}\left[\mathrm{KF}‐18C6\right]\left[\mathrm{KF}‐18C6‐H2O\right]}{\left[\mathrm{KF}‐18C6‐\mathrm{HF}\right]}$$

The least equilibrium is the addition of water to KOH-18C6:

18 $$K_{4}=\frac{\left[\mathrm{KOH}‐18C6‐H_{2}O\right]}{\left[\mathrm{KOH}‐18C6\right]\left[H_{2}O\right]}$$

and the concentration of KOH-18C6-H_2_O complex can be written as

19 $${\left[\mathrm{KOH}‐18C6‐H_{2}O\right]=K}_{4}\left[\mathrm{KOH}‐18C6\right][H_{2}O]$$

Because K_3_ has a small value (calculated Δ*G* = 12.2 kcal mol^–1^), the concentration of KOH-H_2_O remains very low and can be calculated from eq 17. In the same way, the concentration of KOH-18C6-H_2_O can be calculated from eq 19. The rate constants for all of the pathways in Schemes 4 and 5 can be calculated from the free energy profiles. Once the concentrations of these species are defined, the last step is the calculation of the initial reaction rate leading to the products RF, ROH, and PE2. However, the kinetics of alcohol formation depends on the concentration of the KHF_2_(18C6) species, which is generated from the equilibrium for the formation of KOH-18C6 and from the E2 reactions. Thus, the KHF_2_(18C6) species is present in low concentration at the beginning of the reaction, and its concentration increases as the reaction advances, changing the yield of ROH product. A consequence of this behavior is that more E2 products inhibit the formation of the alcohol product. It is worthwhile to notice that while an alcohol product was observed from fluorination of primary alkyl bromide, no alcohol was observed from secondary alkyl bromide, in line with a substantially more E2 product observed for secondary substrate.^31^

Aimed to analyze quantitatively if the equilibrium given by eq 16 and reactions via TS3-18C6 and TS3-18C6-H_2_O can be the source of the alcohol product, we have calculated the selectivity of alcohol formation as a function of the concentration of KHF_2_(18C6) species, as reported in Figure 6. Using the theoretically calculated value of K_3_ (Figure 6a) results in a prediction of very small formation of the alcohol, considering a reasonable concentration of the KHF_2_(18C6) species (0.01 M) as the reaction advances. However, it is possible to notice that the formation of alcohol is highly dependent on the value of [KHF_2_(18C6)]. Thus, an error of 3 kcal mol^–1^ in the free energy profile of Scheme 3 can lead to a variation in K_3_ by a factor of 10^2^. This effect was analyzed, and we can see in Figure 6b that the formation of alcohol product is substantially increased in all concentrations, reaching 4.9% selectivity when [KHF_2_(18C6)] = 0.01 M. Consequently, considering the uncertainty in the calculated free energy profile, which can be optimistically estimated within 3 kcal mol^–1^ for this complex system, the present proposed mechanism in Schemes 3–5 can explain the formation of alcohol side product. This finding is not important only for the water present in the medium. It has implications for other hydrogen-bonding donor species, such as bulky alcohols and diarylureas, also used for inducing S_N_2 selectivity.

**Figure 6:**
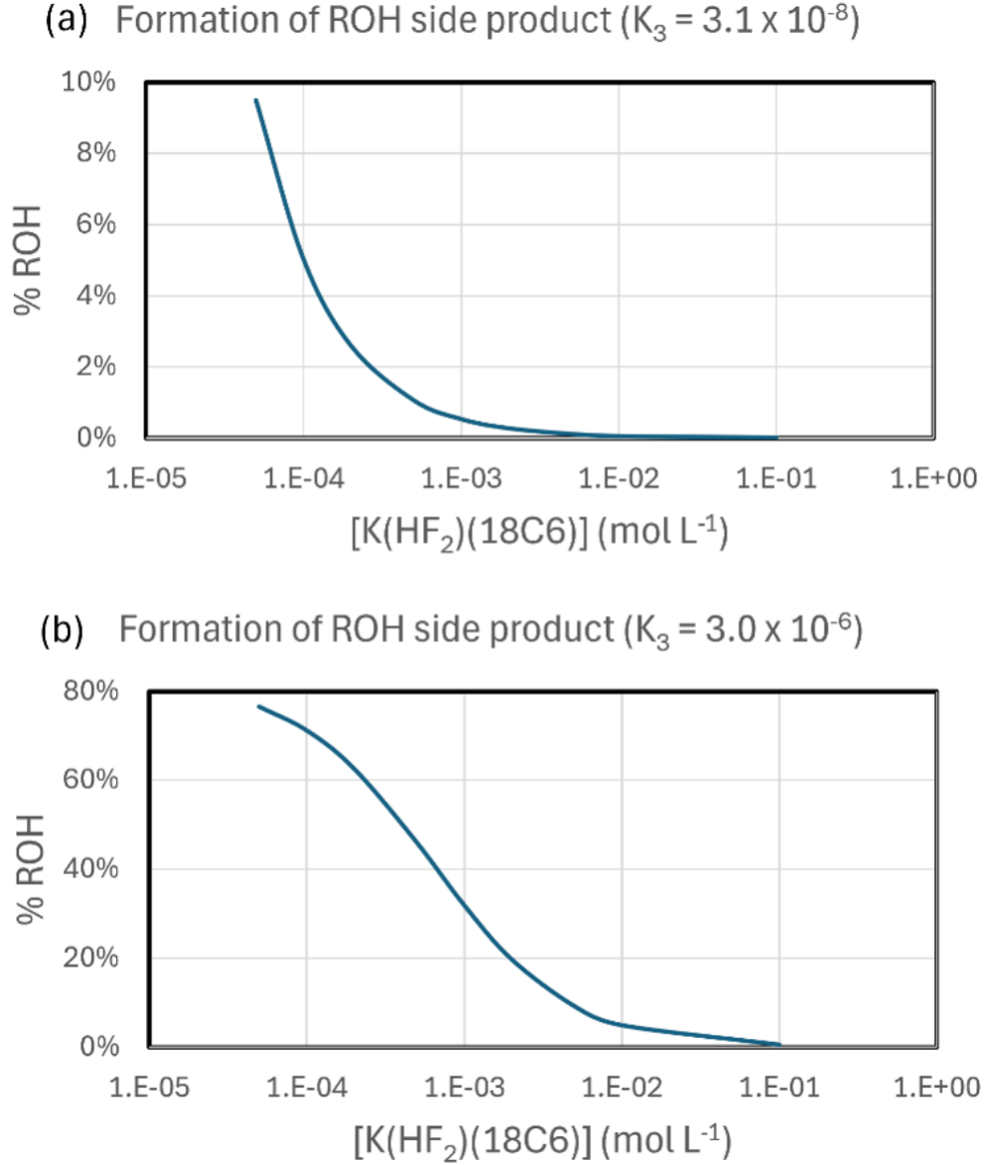
Calculated selectivity of alcohol formation as a function of the concentration of [K­(HF2)­(18C6)], considering the free energy profiles of Schemes 3–5, at 82 °C, 0.25 M of substrate, 0.25 M of crown ether, and 0.75 M of water. The simulations consider two values of K_3_ constant: in (a) the theoretically calculated value and in (b) using a value inside the uncertainty range of Δ*G*.

## 3 Conclusions

A detailed exploration of the mechanism of alcohol side product formation in nucleophilic fluorination of a primary alkyl bromide substrate with KF mediated by crown ether was done by using computational modeling and experiments. Our findings indicated that direct reaction with water molecules is kinetically very slow, and the mechanism must take place via the formation of KHF2­(18C6) and KOH­(18C6) species at low concentration. It occurs because the formation of the HF_2_^–^ ion makes the equilibrium constant for deprotonation of water and the formation of KOH­(18C6) species more favorable. In addition, the KOH­(18C6) species is much more reactive than the KF­(18C6) species toward the primary alkyl bromide substrate. Consequently, the reaction involving KOH­(18C6) achieves a sufficiently high reaction rate to compete with nucleophilic fluorination, thereby accounting for the formation of the alcohol side product.

## 4 Theoretical Methods

The free energy profile in the solution phase for this reaction system was obtained from a composite scheme. Initially, the structures were fully optimized using the X3LYP functional^45–47^ and the def2-SVP basis set^48^ (ma-def2-SVP for O, F, Br).^49^ The CPCM continuum method^50–53^ with the parameters recently reported^54^ for acetonitrile was included in the optimizations, using the van der Waals surface. These initial optimizations were followed by harmonic frequency calculations at the same level of theory to obtain vibrational, rotational, and translational contributions to the free energy (*G_n_*). For obtaining more reliable electronic energies (*E*_el_), single-point energy calculations were done using the ωB97M-V functional^55^ with the def2-TZVPP basis set (mas-def2-TZVPP for O, F, Br), which has a very good performance for chemical reactions.^56^ The final solvation free energy was also obtained by a single-point calculation using the X3LYP functional and the more reliable SES surface^52,57^ (Δ*G*_solv_). A refinement was also made for the vibrational contribution to the free energy involving very low frequencies.^31^ The transformation of the low harmonic vibrational modes into internal rotation was also included in the calculations of the thermodynamic properties. However, we have included this transformation for both enthalpy and entropy, as reported in our previous study,^31^ instead of just making this transformation for entropy as proposed by Grimme.^58^ Recent study supports this approach as more reliable.^59^ Finally, correction of the standard state of 1 atm (gas phase) to 1 mol L^–1^ (solution) was also included in the free energy. The final free energy in the solution phase for each species is given by

20 $$G_{sol}\left(X\right)=E_{el}\left(X\right)+G_{n}\left(X\right)+\Delta G_{solv}\left(X\right)+1.89kcal.{mol}^{-1}$$

An initial conformational analysis was performed for the alkyl bromide reactant. We found 4 conformations that differ by less than 0.7 kcal mol^–1^. Aimed to avoid a large number of conformations in the transition states, we used only the linear conformation in this study. All the calculations were made with the ORCA 5.0.3 program.^60,61^

## 5 Experimental Section

All of the reagents were obtained commercially and used without further purification. The experimental procedures to carry out the reactions are described in the SI file. Thin layer chromatography was performed on TLC plates (silica gel 60 F254) and visualized by employing a UV lamp (254 nm). The ^1^H and ^13^C NMR spectra were recorded at 500 and 125 MHz, respectively, on a Bruker Avance III 500 MHz. Chemical shifts for ^1^H and ^13^C NMR were reported as δ (parts per million (ppm)) relative to the signals of CDCl_3_ at 7.26 ppm (singlet) and 77 ppm (triplet). Tetramethylsilane (TMS) was established as an internal reference. NMR chemical shifts are reported employing the peak abbreviation pattern: s, singlet; d, doublet; dd, double doublet; t, triplet; dt, double triplet; qui, quintet; dqui; double of quintets; m, multiplet.

## Supplementary Material



## Notes

General procedures for control experiments of hydrolysis reactions; ^1^H NMR spectra of fluorination and hydrolysis experiments; table with experimental data of the reactions showing product yields; table of electronic energies of all optimized structures; *XYZ* coordinates of all optimized structures; additional references (PDF)
