# Supplementary material for: Revealing the Mechanism of Alcohol Side Product Formation in Crown Ether-Mediated Nucleophilic Fluorination Using Acetonitrile as Solvent
Source: ACS Omega. 2025 Jul 16;10(29):32372–83. doi: 10.1021/acsomega.5c04699 (PMC12311644; doi:10.1021/acsomega.5c04699)
Supplement: Supplementary file 1 [file ao5c04699_si_001.pdf]

Revealing the Mechanism of Alcohol Side Product Formation in Crown Ether Mediated Nucleophilic Fluorination Using Acetonitrile as Solvent

Eloah P. Ávila[1], Mauro V. de Almeida[2] and Josefredo R. Pliego Jr.[1]*

[1]Departamento de Ciências Naturais, Universidade Federal de São João del-Rei, São João del-Rei, MG, 36301-160, Brazil.

[2]Departamento de Química, Universidade Federal de Juiz de Fora, Campus Universitário, Martelos, Juiz de Fora, MG, 36036-330, Brazil

* pliego@ufsj.edu.br

Table of contents

1. General procedures of control experiments of hydrolysis reactions 3

2. NMR spectra of experiments 5

2.1. Identification of ROH and ROR species 5

2.2. Hydrolysis reactions under [18C6-KF] conditions 7

2.3. Hydrolysis reactions under [18C6-KOH] conditions 10

3. Theoretical data 17

4. XYZ coordinates 18

5. References 38

1. General procedures of control experiments of hydrolysis reactions

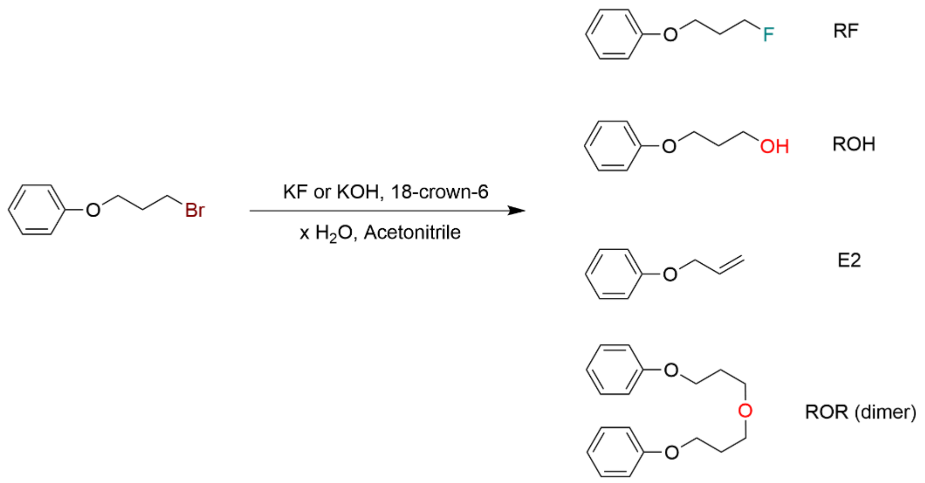

Scheme S1: General reaction conditions.

Control experiment 1: Alkylbromine (0.5 mmol) and H_2O (1.5 mmol) were added in a vial containing 2 mL of acetonitrile. The reaction was stirred at 82 °C for 24 h and monitored by TLC. The solvent was removed under reduced pressure. The crude reaction was submitted to ^1H NMR measurements to obtain the conversion rates (Table S1).

Control experiment 2: Alkylbromine (0.5 mmol), 18-crown-6 (0.5 mmol) and KOH (1.0 mmol) were added in a vial containing 2 mL of acetonitrile. The reaction was stirred at 50 °C, then some aliquots were taken, in 2 h, 4 h and 6 h and the solvent was removed under reduced pressure. The crude reactions were submitted to ^1H NMR measurements to obtain the yields (Table S1). The NMR data of isolated products are described in the literature.[1]

Control experiment 3: Alkylbromine (0.5 mmol), 18-crown-6 (0.5 mmol), KOH (1.0 mmol) and H_2O (1.5 mmol) were added in a vial containing 2 mL of acetonitrile. The reaction was stirred at 50 °C, then some aliquots were taken, in 2 h, 4 h and 6 h and the solvent was removed under reduced pressure. The crude reactions were submitted to ^1H NMR measurements to obtain the yields (Table S1).

Control experiment 4: Alkylbromine (0.5 mmol), KOH (1.0 mmol) and H_2O (1.5 mmol) were added in a vial containing 2 mL of acetonitrile. The reaction was stirred at 82 °C, then some aliquots were taken, in 3 h and 20 h and the solvent was removed under reduced pressure. The crude reactions were submitted to ^1H NMR measurements to obtain the yields (Table S1).

2. NMR spectra of experiments

2.1. Identification of ROH and ROR species

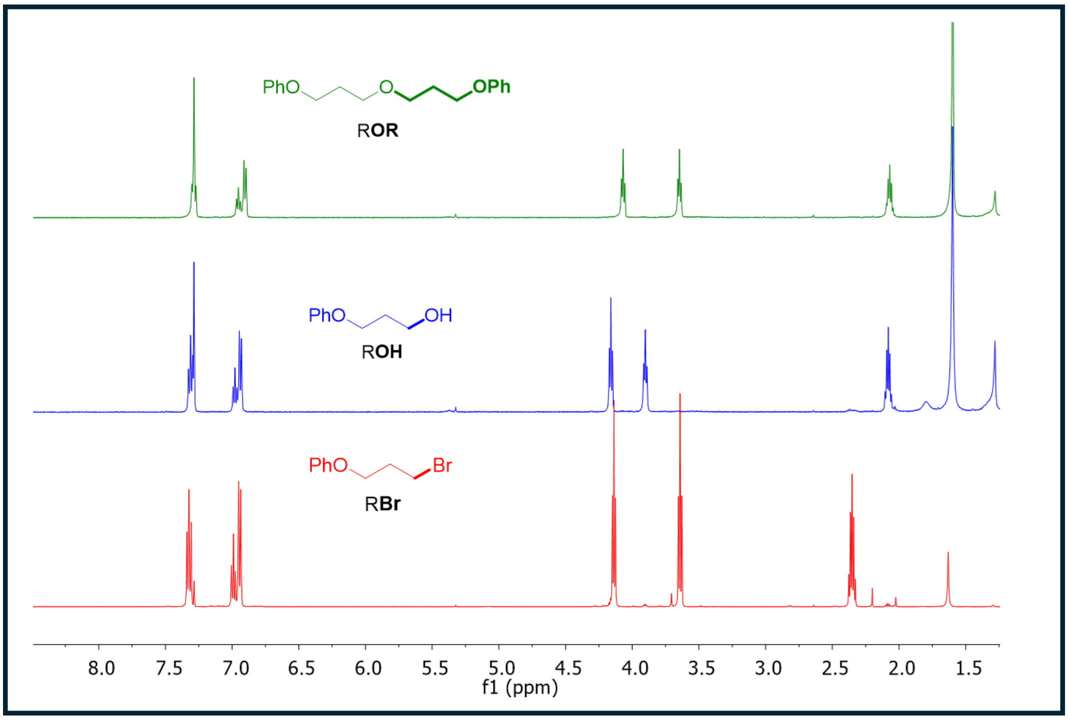

Figure S1. ^{1}H NMR ($CDCl_3$, 500 MHz) full spectra of dimer (ROR), alcohol (ROH) and starting material (RBr).

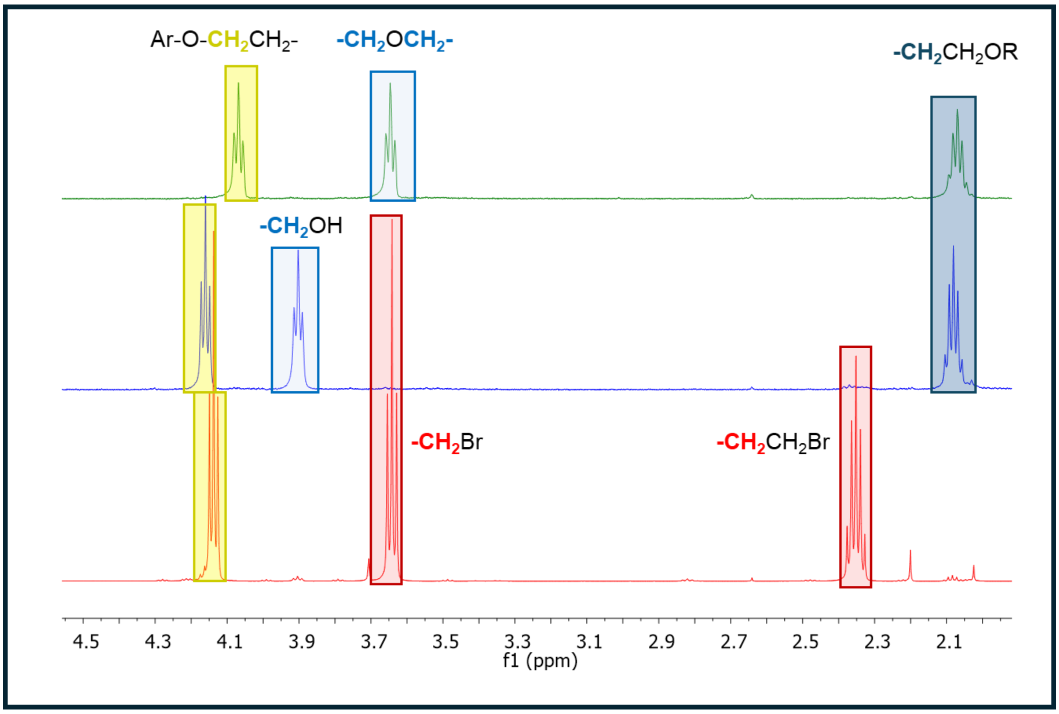

Figure S2. ^{1}H NMR ($CDCl_3$, 500 MHz) spectra (ranging from 4.5 to 1.9 ppm) of dimer (ROR), alcohol (ROH) and starting material (RBr).

2.2. Hydrolysis reactions under [18C6-KF] conditions

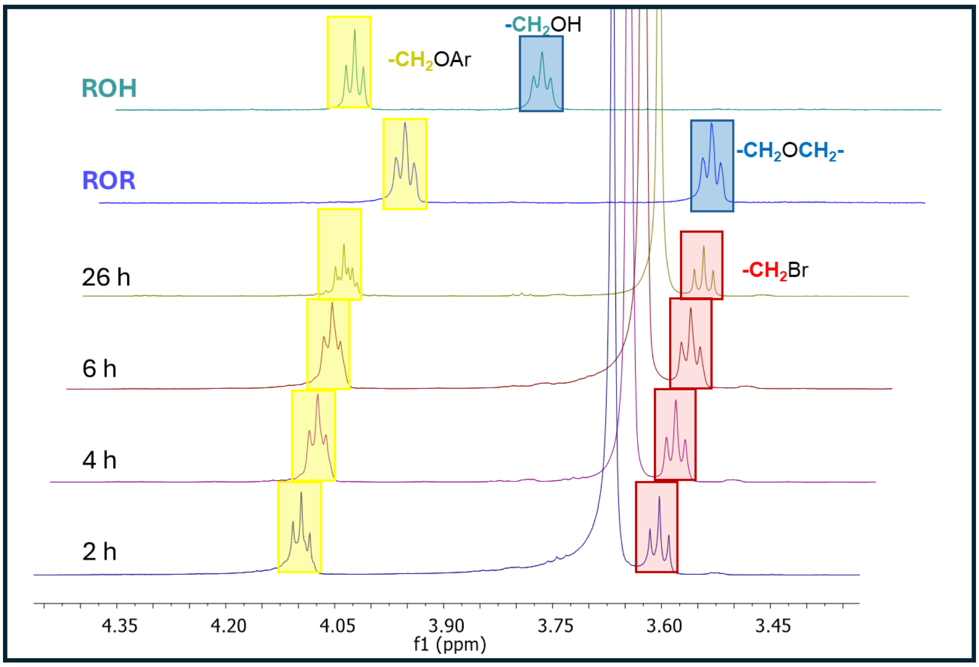

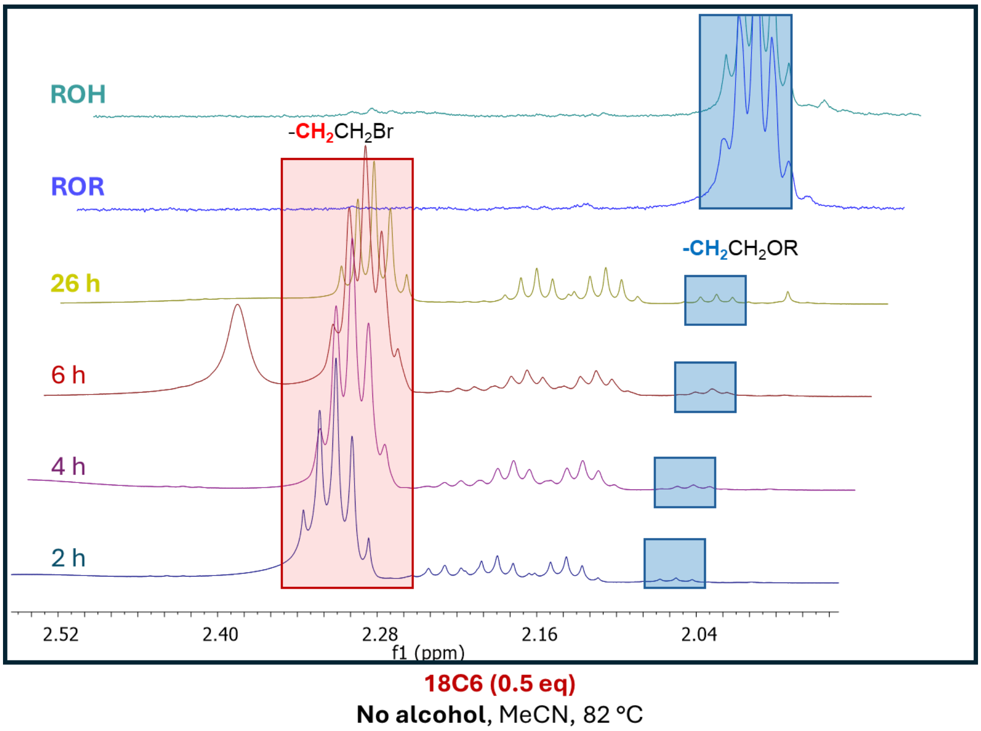

Figure S3. ^{1}H NMR ($CDCl_3$, 500 MHz) spectra of fluorination reaction (50 mol % of 18C6 and 2 eq of KF) in 2, 4, 6 and 26 h – Entries 1-4.

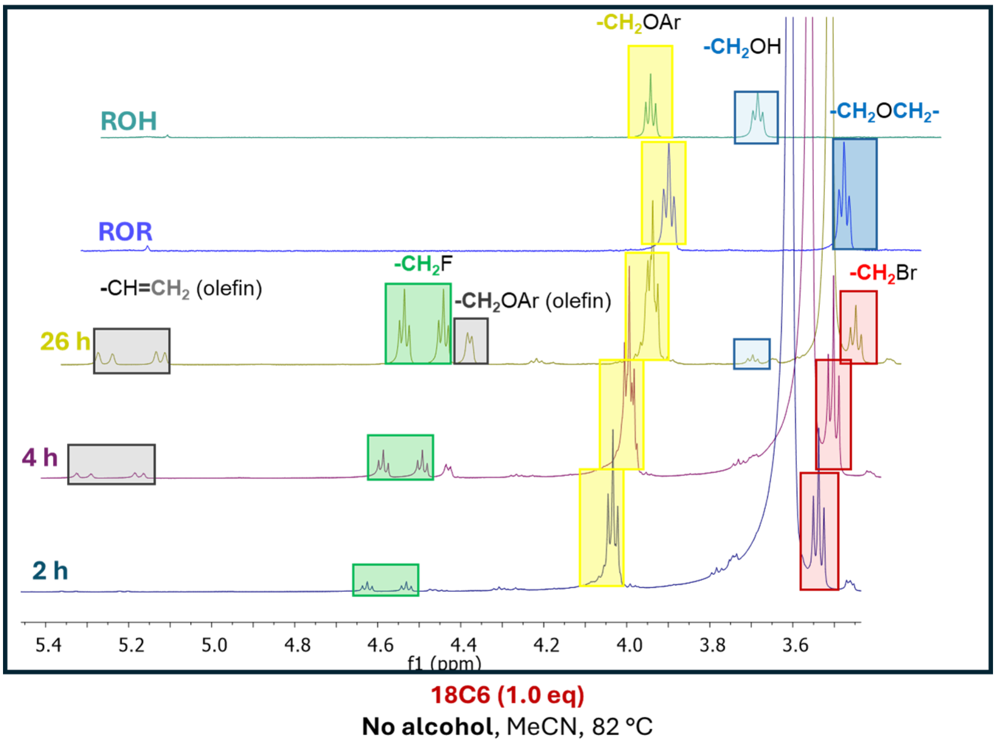

Figure S4. ^{1}H NMR ($CDCl_3$, 500 MHz) spectra of fluorination reaction (1 eq of 18C6 and 2 eq of KF) in 2, 4, 6 and 26 h – Entries 5-7.

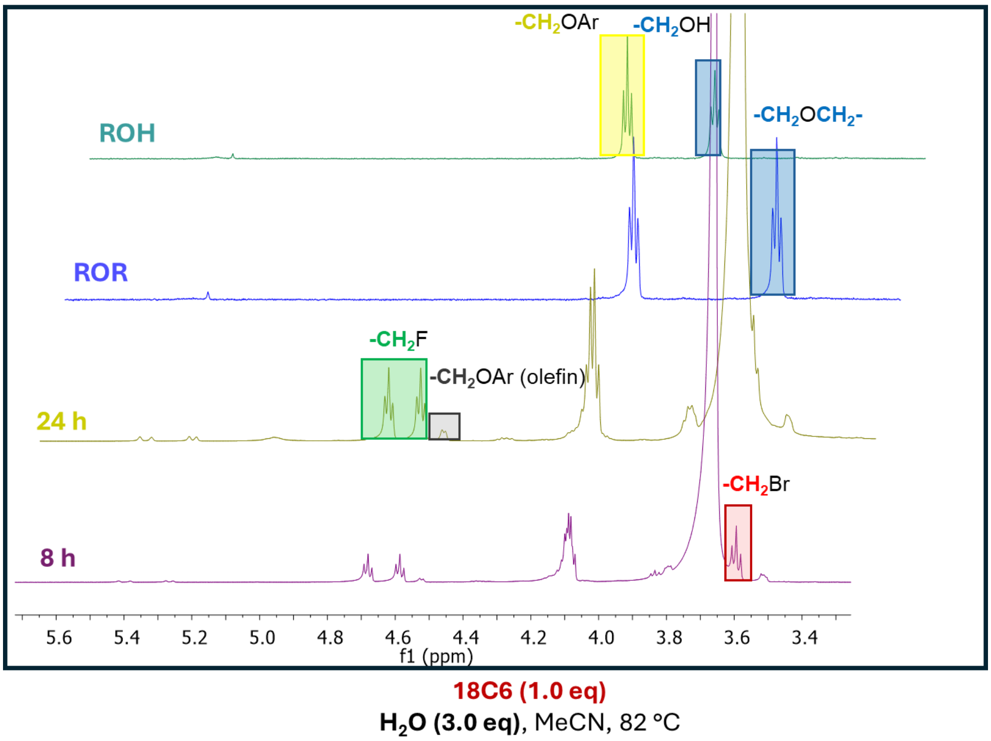

Figure S5. ^{1}H NMR ($CDCl_3$, 500 MHz) spectra of fluorination reaction (1 eq of 18C6, 3 eq of H_2O and 2 eq of KF) in 8 and 26 h – Entries 8-9.

2.3. Hydrolysis reactions under [18C6-KOH] conditions

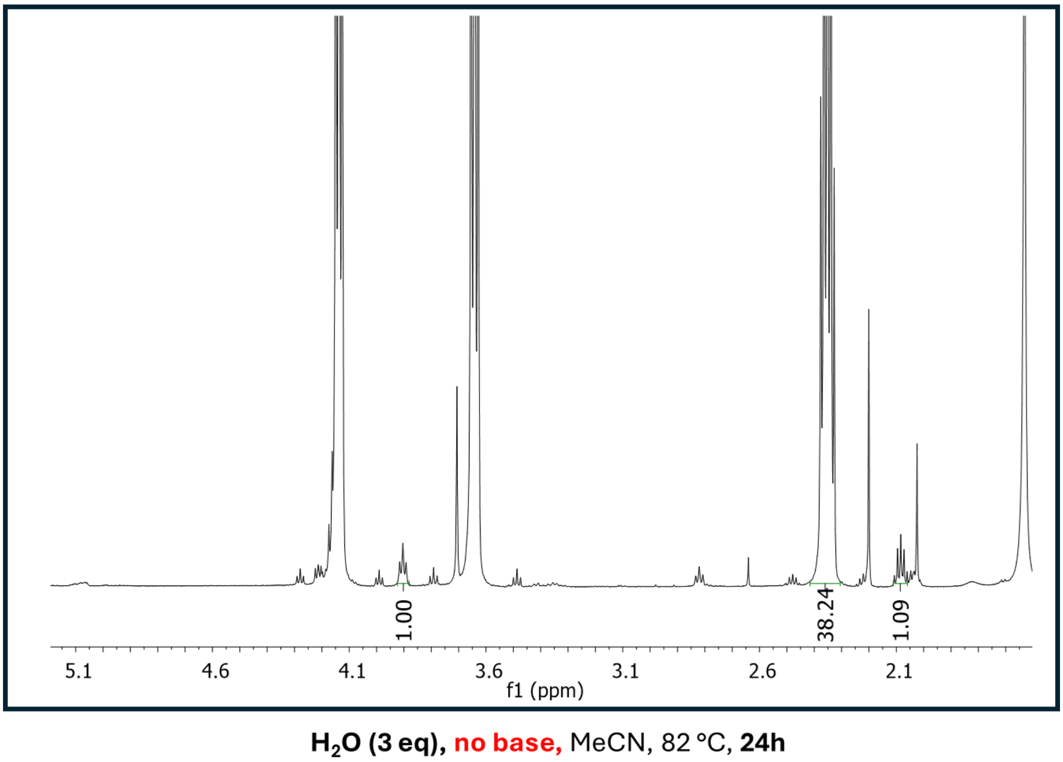

Figure S6. ^{1}H NMR ($CDCl_3$, 500 MHz) spectra of **Control experiment 1**, after 24 h – Entry 10.

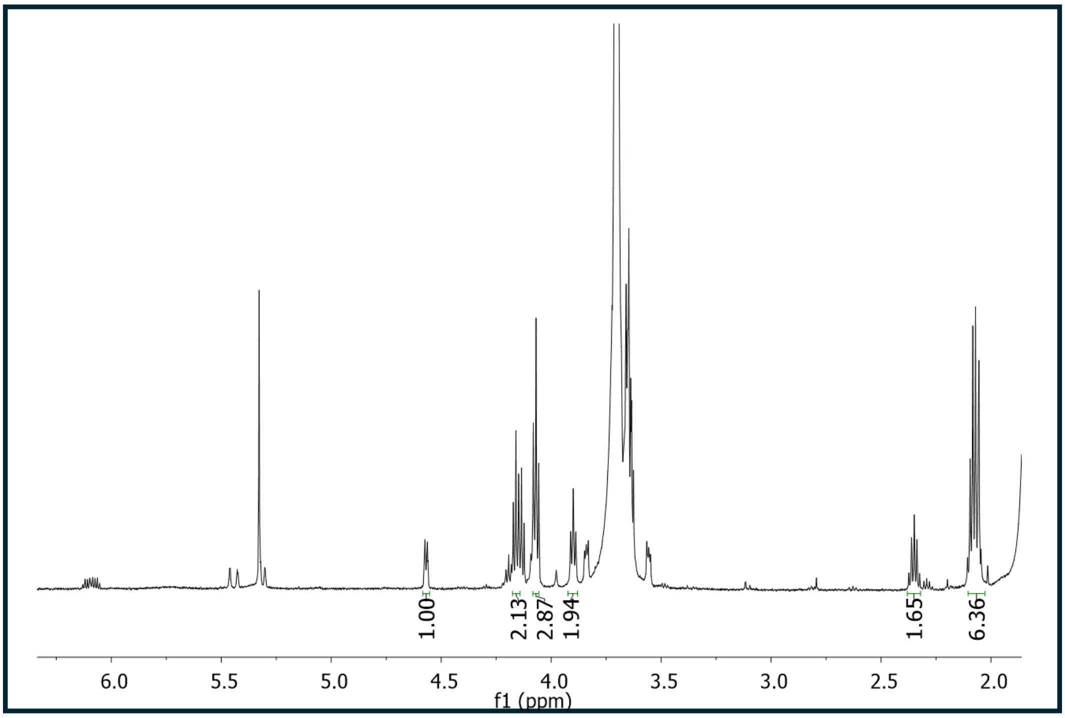

18C6 (1 eq)
No alcohol, KOH, MeCN, 50°C, **2h**

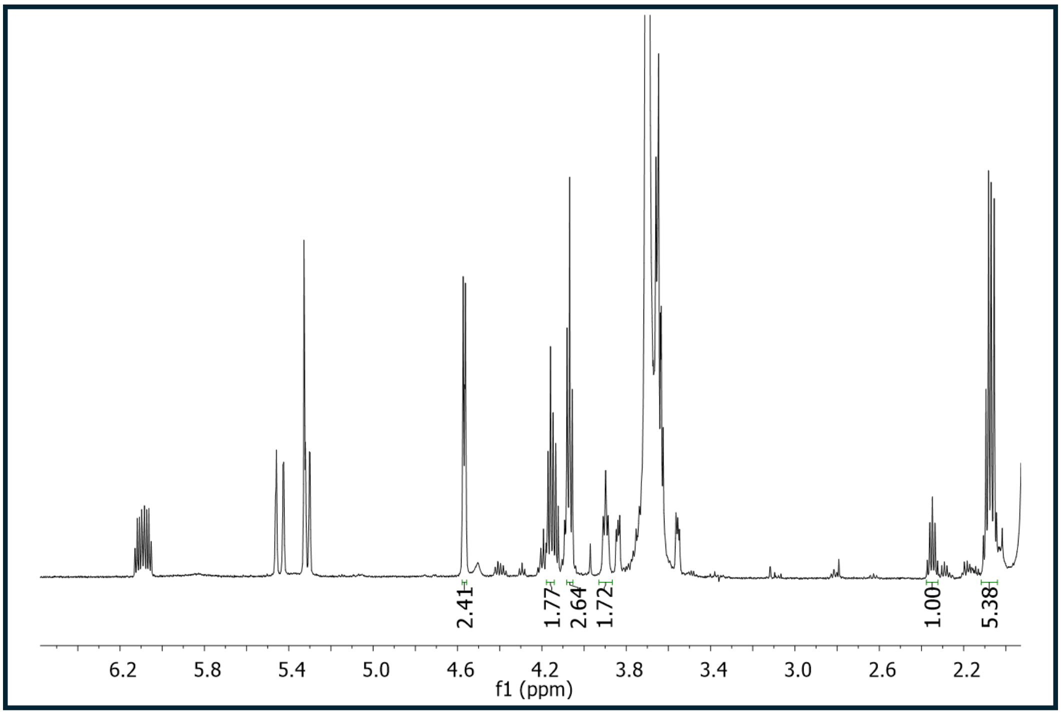

18C6 (1 eq)
No alcohol, KOH, MeCN, 50°C, **4h**

Figure S7. ^{1}H NMR ($CDCl_3$, 500 MHz) spectra of **Control experiment 2**, after 2 h and 4 h – Entries 11-12.

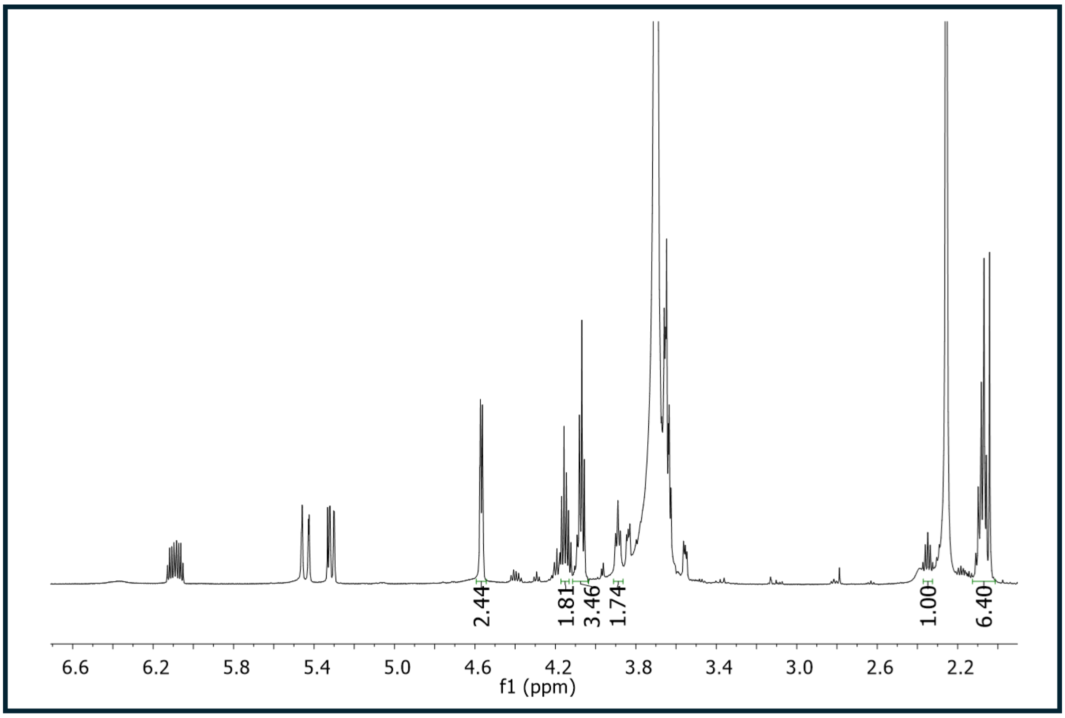

18C6 (1 eq)

No alcohol, KOH, MeCN, 50°C, **6h**

Figure S8. ^{1}H NMR ($CDCl_3$, 500 MHz) spectra of **Control experiment 2**, after 6 h – Entry 13.

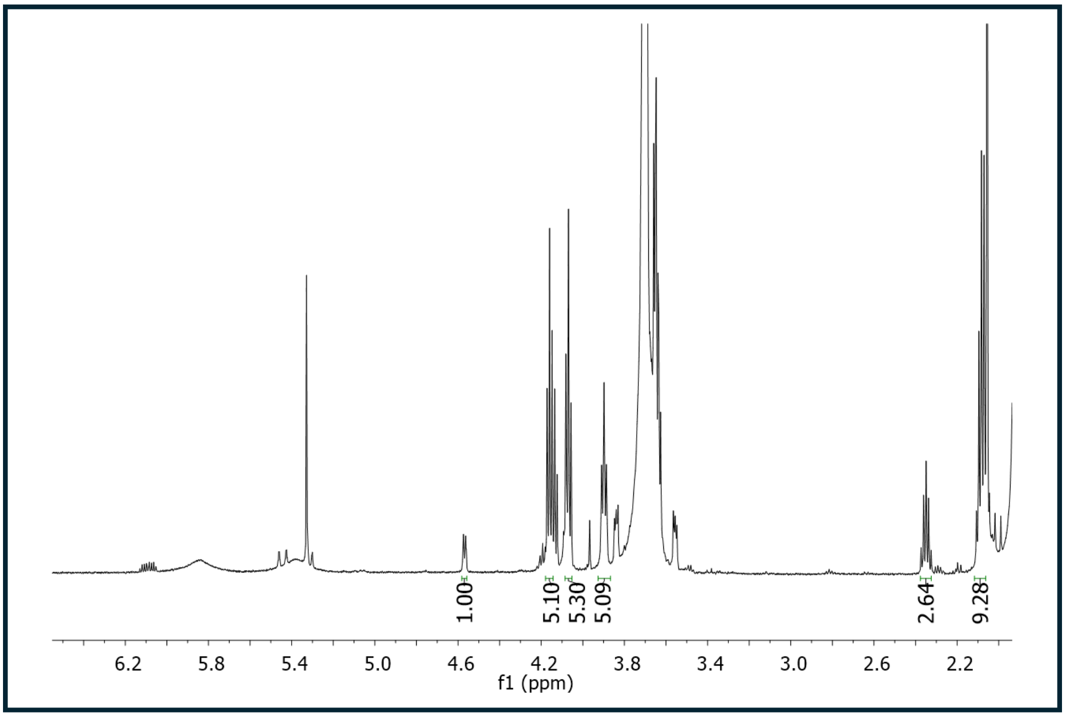

18C6 (1 eq)
H_2O (3 eq), KOH, MeCN, 50°C, **2h**

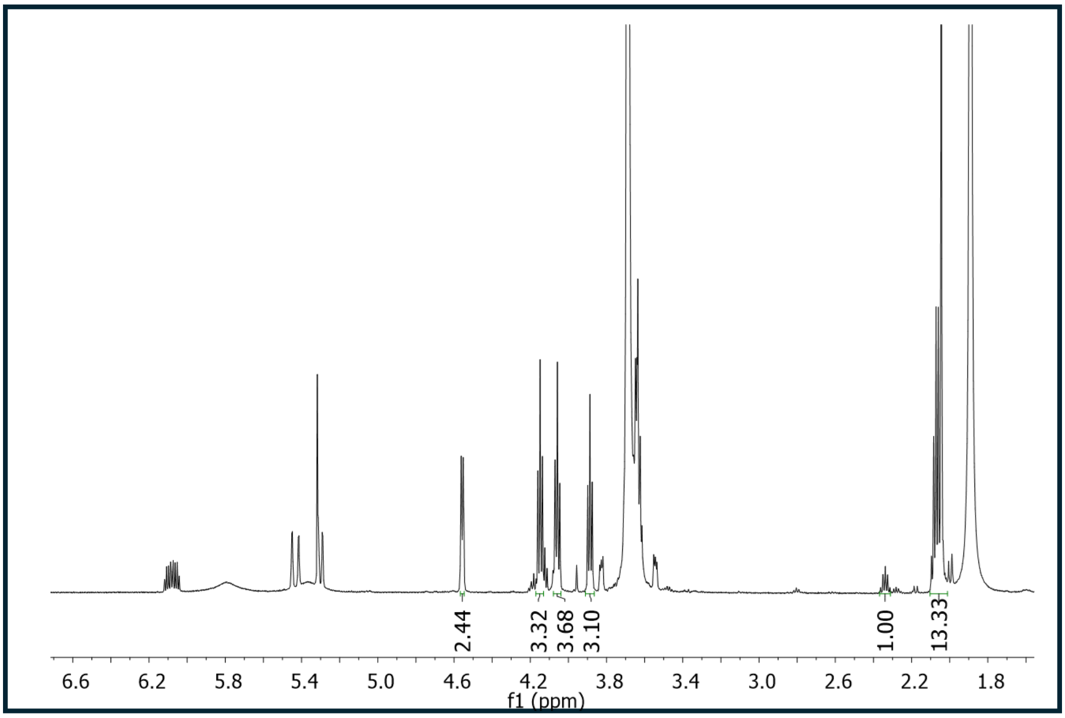

18C6 (1 eq)
H_2O (3 eq), KOH, MeCN, 50°C, **4h**

Figure S9. ^{1}H NMR ($CDCl_3$, 500 MHz) spectra of **Control experiment 3**, after 2 h and 4 h – Entries 14-15.

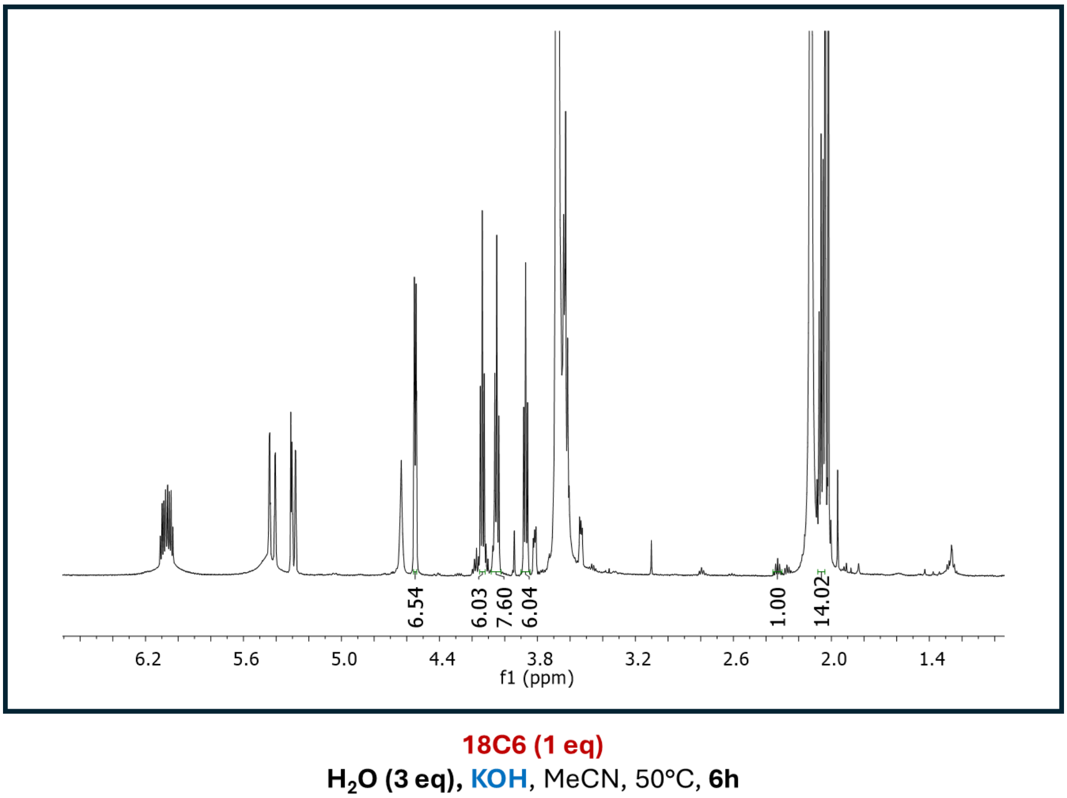

Figure S10. ^{1}H NMR ($CDCl_3$, 500 MHz) spectra of **Control experiment 3**, after 6 h – Entry 16.

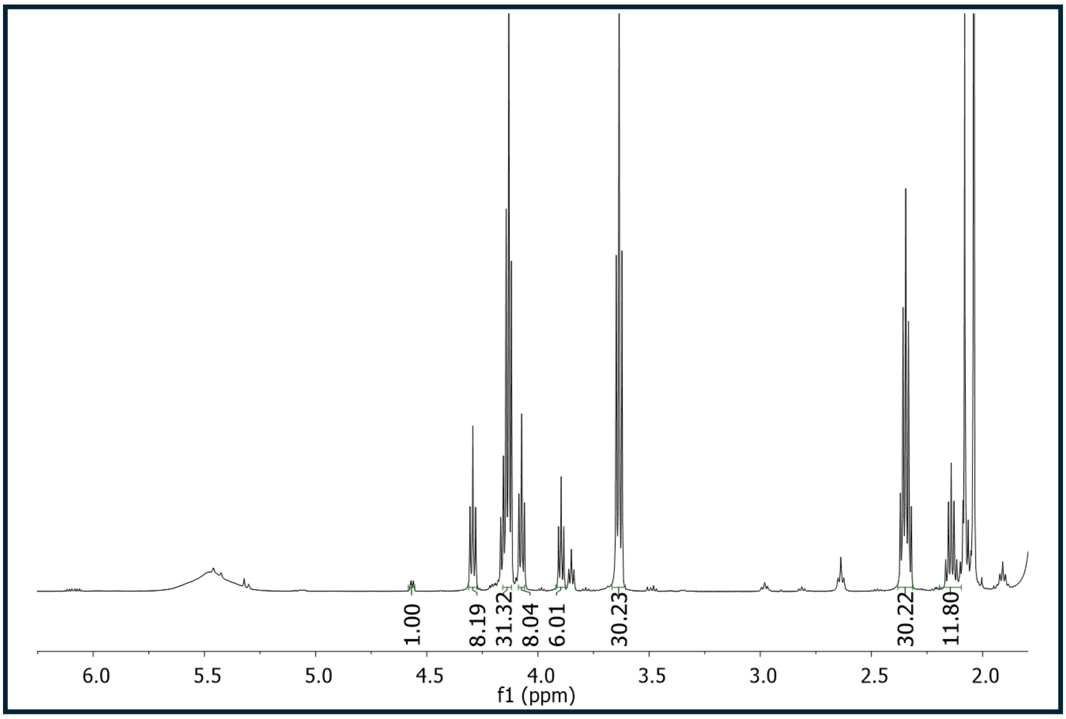

H_2O (3 eq), KOH, MeCN, 82°C, **3h**

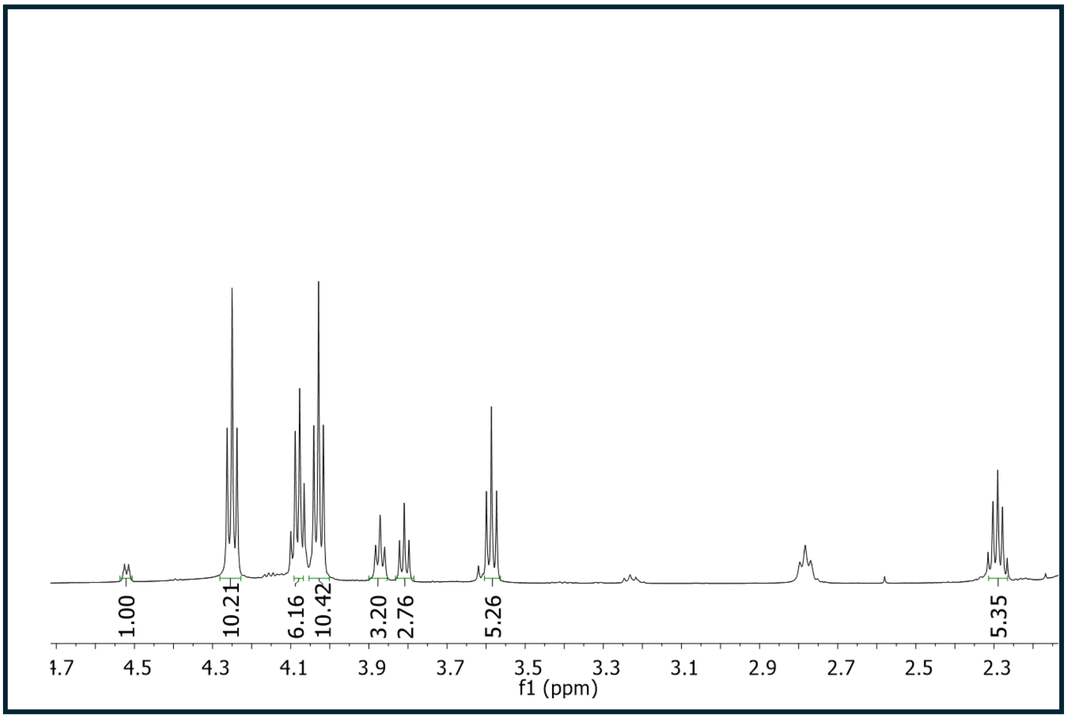

H_2O (3 eq), KOH, MeCN, 82°C, **20h**

Figure S11. ^{1}H NMR ($CDCl_3$, 500 MHz) spectra of **Control experiment 4**, after 3 h and 20 h – Entries 17-18.

Table S1: Experimental data of the reactions with the primary alkyl bromide.

Entry	Time / h	Salt	18C6 / eq	H2O / eq	Solvent	Temperature / °C	Total conversion	RF	ROH	ROR (dimer)	E2
1	2	KF	0.5	-	MeCN	82	21.8%	16.6%	2.4%	0.0%	2.8%
2	4	KF					25.5%	19.5%	2.1%	0.0%	3.9%
3	6	KF					27.8%	20.0%	4.6%	0.0%	5.0%
4	26	KF					41.2%	28.8%	7.3%	0.0%	7.8%
5	2	KF	1.0	-	MeCN	82	31.0%	20.1%	8.1%	0.0%	2.7%
6	4	KF					36.5%	21.2%	10.6%	0.0%	4.8%
7	26	KF					80.0%	54.9%	12.5%	0.0%	12.5%
8	8	KF	1.0	H2O / 3	MeCN	82	62.0%	51.0%	6.9%	0.0%	4.1%
9	24	KF					79.6%	60.0%	14.8%	0.0%	4.8%
10	24	-	-	H2O / 3	MeCN	82	2.5%	-	2.5%	0.0%	0.0%
11	2	KOH	1.0	-	MeCN	50	72.6%	-	32.2%	23.8%	16.6%
12	4	KOH					84.5%	-	26.7%	20.5%	37.4%
13	6	KOH					85.3%	-	23.9%	25.5%	35.9%
14	2	KOH	1.0	H2O / 3	MeCN	50	76.8%	-	44.8%	23.3%	8.8%
15	4	KOH					88.4%	-	38.6%	21.4%	28.4%
16	6	KOH					94.2%	-	34.7%	21.9%	37.7%
17	3	KOH	-	H2O / 3	MeCN	82	26.2%	-	20.0%	3.7%	2.4%
18	20	KOH					70.9%	-	56.5%	8.9%	5.5%

3. Theoretical data

Table S2: Single point electronic energies of the optimized structures using the def2-TZVPP (ma-def2-TZVPP for O, F and Br) basis set.[a]

Species	ωB97M-V
KF(g)	-699.810199
KBr(g)	-3173.995485
18C6	-922.989727
KF-18C6	-1622.857547
KBr-18C6	-4097.052179
H2O	-76.43592618
HF	-100.463195
KBr-18C6-H2O	-4173.482779
KF-18C6-H2O	-1699.327316
KF-18C6-2H2O	-1775.786549
KF-18C6-HF	-1723.377353
KOH	-675.75424
KOH-18C6-H2O	-1675.269231
KOH-18C6-2H2O	-1751.728466
KOH-18C6	-1598.787134
PE2	-424.1499405
RBr	-2998.794500
RF	-524.6382441
ROH	-500.6081047
TS1-18C6	-4621.655353
TS1-18C6-H2O	-4698.114284
TS1-H2O	-3075.156801
TS2-18C6	-4621.649077
TS2-18C6-H2O	-4698.100664
TS3-18C6-H2O	-4674.061388
TS3-18C6	-4597.599026
TS4-18C6-H2O	-4674.056004
TS4-18C6	-4597.596061

a – Units in Hartree.

4. XYZ coordinates

H2O

```
3
Coordinates from ORCA-job geom
  O   -6.65345391057039      0.99254467040993     -0.01767508514178
  H   -5.68703212538341      1.00242537993510      0.02459452749928
  H   -6.93463396404619      1.20310994965497      0.88363055764251
```

HF

```
2
Coordinates from ORCA-job geom
  F   2.24072149307701      0.00000000000000      0.00000000000000
  H   3.17314850692299      0.00000000000000      0.00000000000000
```

KBr-18C6-H2O

```
47
Coordinates from ORCA-job geom
  C   -3.22853031280899     -2.63732717599847     -3.74091015849858
  O   -1.90438611473431     -3.10883765627057     -3.55726092563551
  C   -4.14064453901097     -3.28074188870849     -2.72393401720880
  C   -0.98066039718649     -2.60612905478349     -4.50823517601843
  C   0.37550436762784     -3.23074913289114     -4.28069761267715
  O   0.87376825386993     -2.84337556422119     -3.01208605583140
  O   -3.76130468997593     -2.87736025572101     -1.41963841254160
  C   2.17210538675291     -3.33988343617465     -2.73386985994867
  C   2.62922130909326     -2.85690456175454     -1.37785959717095
  C   -4.57524351767099     -3.42387776015823     -0.39600719458849
  C   -4.09619382959344     -2.94667202243620      0.95453971772313
  O   -2.80078424748815     -3.46113823223072      1.21584446651372
  O   1.80584984561585     -3.41298790412929     -0.36748855248498
  C   -2.31730265912109     -3.15181200661626      2.51133345846802
  C   2.21743395420038     -3.08528766530126      0.94768720742183
  C   1.29300385456563     -3.73801595546747      1.94759537443111
  C   -0.95815153702008     -3.77881235257476      2.71357187098904
  O   -0.01278689209370     -3.18839951108631      1.83089481552345
  H   -3.58998920012831     -2.89268845949347     -4.75509650316075
  H   -3.25916918557037     -1.53525772499235     -3.63869583210125
  H   -5.18276951753062     -2.97356760156227     -2.93435821466468
  H   -4.08722507506458     -4.38300526739719     -2.81093889387439
  H   -0.91112560518556     -1.50406202233712     -4.42673720436891
  H   -1.31529095114952     -2.84827756297143     -5.53474907937493
  H   1.06052534972648     -2.89414606415896     -5.08197803885948
  H   0.30142786931769     -4.33361886516082     -4.33992298123957
  H   2.88954556246560     -2.98424857627017     -3.49772315599680
  H   2.16962456740238     -4.44651137423308     -2.76022450409451
  H   3.68108167683766     -3.16775605351741     -1.22989871550995
  H   2.59243024532572     -1.75147770423611     -1.33294965267943
  H   -4.54634485460411     -4.52965711478635     -0.43812009999326
  H   -5.62674656584549     -3.10666997047919     -0.53241849034121
  H   -4.80959515901193     -3.29781391586962      1.72417036134222
  H   -4.08018331173588     -1.84043808085930      0.98638623044927
  H   -3.00457080311234     -3.54967269765166      3.28209497622609
  H   -2.25942089687504     -2.05486263534032      2.64525924295935
```

```
  H   3.24640465699106     -3.44939977057180      1.13104118737938
  H   2.22001715791111     -1.98715036590957      1.08515237996839
  H   1.68720612562033     -3.56913217224331      2.96703853276722
  H   1.25551894315223     -4.82934167862907      1.77182496545026
  H   -0.64567104684586     -3.62672828437481      3.76353972960520
  H   -1.01245813571688     -4.86777513552256      2.52776814585505
  K   -0.98003724577900     -2.83538606638677     -0.88713322796707
  Br  -0.05752311174797      2.51992134293958      1.61511756927644
  O   -0.50322906808326     -0.61746412608859      0.56683105750997
  H   -0.36527531867936      0.30965954078070      0.85608297677110
  H   -0.22481533710563     -1.17674945615233      1.31069789020062
```

KBr-18C6-HF

```
46
Coordinates from ORCA-job geom
  C   -3.09597040321293     -2.41226667079171     -3.60801620303359
  O   -1.78868444090892     -2.94005750734171     -3.46659379791282
  C   -4.02253607193686     -3.08575640982796     -2.62509121802522
  C   -0.86464798275863     -2.43042969716214     -4.41254483262915
  C   0.46858700256976     -3.11838394401027     -4.24294598423497
  O   1.01049859631260     -2.79858765185758     -2.97518735305433
  O   -3.62814634084977     -2.76906492498147     -1.30296539388701
  C   2.28210118229086     -3.37328963863938     -2.73506009991540
  C   2.77050948879410     -2.97219080325965     -1.36363714292933
  C   -4.44863628916179     -3.35916352914718     -0.31083679608699
  C   -3.94271568562200     -2.98244010419129      1.06049628460367
  O   -2.65372684717979     -3.53794425640405      1.25943720303765
  O   1.91640367745724     -3.52245305187811     -0.37607913438302
  C   -2.14365796494535     -3.33834203931087      2.56675956763210
  C   2.34871803576324     -3.28329503176647      0.95191335438884
  C   1.42873775985774     -3.99108153026826      1.91752973221674
  C   -0.80972670578813     -4.03246501910517      2.70368443233813
  O   0.12976935800183     -3.43521082921758      1.82924736898830
  H   -3.47349312290958     -2.59240383679824     -4.63260750965430
  H   -3.08707017466498     -1.31841341127101     -3.43733539870183
  H   -5.05649175824002     -2.73784062209818     -2.81272145026409
  H   -4.00086639579321     -4.18234531331484     -2.77546825142694
  H   -0.74804258567167     -1.33733486625640     -4.28154825331131
  H   -1.22945645499218     -2.61162842289497     -5.44153734335438
  H   1.14859154993620     -2.78128179337871     -5.04871239327116
  H   0.34600538563778     -4.21440201547391     -4.33951927653776
  H   3.01158061735251     -3.02359283699327     -3.49064767220725
  H   2.22166809077734     -4.47628626197426     -2.80702047112681
  H   3.80326394327814     -3.34710235607498     -1.23025979199016
  H   2.79588951824166     -1.86878891320459     -1.27478716729497
  H   -4.44778914083747     -4.46042799960454     -0.42395961152884
  H   -5.49283576337894     -3.00717101519130     -0.41633698864894
  H   -4.64963324589540     -3.37161053758139      1.81779878554824
  H   -3.90598358129399     -1.88101722456617      1.16556243063980
  H   -2.84038200656964     -3.75614763078415      3.31830976580764
  H   -2.03364755587102     -2.25615480634431      2.77291149402653
  H   3.37740459709370     -3.66501668880604      1.09675752556495
  H   2.35823641407684     -2.19602232696739      1.16074671243754
  H   1.82884352320925     -3.87460076421314      2.94307416058526
  H   1.39660290339279     -5.07312204981385      1.68620173680110
  H   -0.46964536950351     -3.94480497543743      3.75344244120919
```

```
  H   -0.91600338176400     -5.10940906564945      2.47038535265619
  K   -0.80808485988113     -2.65799797256617     -0.76393373066576
  Br  -0.55322553959905      0.68745715079242     -0.40058485500223
  F   -3.33753701678902      0.98233540647138      0.75734107520737
  H   -2.44471495802454      0.87419378915599      0.37885869738939
```

KF-18C6-2H2O

```
50
Coordinates from ORCA-job geom
  C   -3.07375497883444     -2.38726170196607     -3.66264787059009
  O   -1.75881440787086     -2.88783058007936     -3.48798342664935
  C   -4.02198485271163     -3.09423296954780     -2.72336428560238
  C   -0.82487547116678     -2.34700801058432     -4.40601881647367
  C   0.53510573530407     -2.96392267913125     -4.17966219985682
  O   1.04712055641972     -2.56179226823322     -2.91580517387563
  O   -3.68797014808234     -2.78048362455722     -1.38577069874727
  C   2.30660420627257     -3.14170070309777     -2.60044049525269
  C   2.72686156716146     -2.73400698283027     -1.20819830088514
  C   -4.55041619345661     -3.35619842261007     -0.42217792160636
  C   -4.08696566881243     -2.97567019712046      0.96397658570094
  O   -2.82359391832050     -3.56956239738414      1.22122574027785
  O   1.84015635379048     -3.30037513934043     -0.26051160127631
  C   -2.34496562110063     -3.32553544191594      2.53273393081499
  C   2.19879510908271     -3.04561276515720      1.08641664788849
  C   1.27583228057429     -3.80564717563302      2.00946551468689
  C   -0.98727914395579     -3.96310436341856      2.70915434398450
  O   -0.04488481603458     -3.31714487492578      1.87175687436473
  H   -3.41238313183687     -2.55771849375927     -4.70245258330210
  H   -3.09340652137511     -1.29693316319735     -3.47268608558275
  H   -5.05483561055594     -2.76651015807736     -2.95072606270977
  H   -3.97013884053781     -4.18855947699039     -2.88439522270576
  H   -0.76964752023179     -1.24747167070332     -4.29339621795291
  H   -1.13882348117499     -2.56420529895095     -5.44500049033427
  H   1.21691174417191     -2.63804722356949     -4.98752601100910
  H   0.46189578840397     -4.06643827284745     -4.21899186940696
  H   3.07323920303159     -2.80443136704477     -3.32316930908038
  H   2.23644994233751     -4.24312559055967     -2.66560261654031
  H   3.75858918124435     -3.09434105155420     -1.03270081705621
  H   2.73443892580218     -1.63100175646814     -1.11629231602429
  H   -4.56100873992651     -4.45806313597591     -0.53010084324802
  H   -5.58556850064543     -2.99088009701144     -0.56710354221898
  H   -4.83431777923607     -3.33493879544199      1.69751426635184
  H   -4.00792945595014     -1.87651353929133      1.05778588654398
  H   -3.03968678496347     -3.75491064088549      3.28045040490861
  H   -2.27828793239114     -2.23662902932275      2.71358586867988
  H   3.23842845052273     -3.37442903197718      1.27598500127124
  H   2.14281475270972     -1.96034927715487      1.29892484576998
  H   1.62781745676289     -3.67531360110816      3.05107019051826
  H   1.31045078698869     -4.88661418103238      1.77283529279529
  H   -0.68554612867890     -3.86911764194400      3.77007933194859
  H   -1.03670690938962     -5.04201790262835      2.46567268664590
  K   -0.93857271614514     -2.59445868906867     -0.73655923335917
  F   -0.94720977394902      0.00826986340909     -0.66517120992619
  O   -2.42606252840925      0.27337362012055      1.42229540470826
  H   -1.84638306159527      0.17191092064771      0.60990761637825
  H   -1.80800525079866      0.31745005870853      2.16202543648511
```

```
  H   0.18710787586511      0.28381748965766     -1.74614278738455
  O   0.89998463570027      0.36386598355105     -2.44071642545076
  H   1.05693133599165     -0.55904855199625     -2.69751743661524
```

KF-18C6-H2O

```
47
Coordinates from ORCA-job geom
  C   -3.15713520931339     -2.44176863567365     -3.60680693357540
  O   -1.84166913033445     -2.94313069809621     -3.46280131158890
  C   -4.07051725619612     -3.12942138876910     -2.62085301359890
  C   -0.92991662006160     -2.43852414141762     -4.42075985270987
  C   0.41686254454917     -3.09558633758535     -4.23248027320444
  O   0.93922876441224     -2.74276766124586     -2.96835527778579
  O   -3.68006202780288     -2.80265701319690     -1.30255112085131
  C   2.23817443543439     -3.24589418797444     -2.71847426896620
  C   2.68359930643403     -2.83282377895256     -1.33589733144012
  C   -4.50211387938837     -3.38114797684315     -0.30788299228071
  C   -3.98977765732926     -3.00836690998809      1.06262938932137
  O   -2.72501583767714     -3.60816996557561      1.27589659448126
  O   1.91019065442253     -3.51148947911518     -0.36079663935753
  C   -2.21315845232490     -3.40998148293235      2.58034829476944
  C   2.26376416057863     -3.16880576181769      0.96817507562637
  C   1.38239322968797     -3.91828242399732      1.93911592676652
  C   -0.86265529632735     -4.07586324086065      2.70350044084759
  O   0.05692342447059     -3.43194811196263      1.84537548208950
  H   -3.53216201449520     -2.63203138975578     -4.63090697640133
  H   -3.17091799430303     -1.34697903952712     -3.43964890334918
  H   -5.11095380690302     -2.80003746405190     -2.80946705111323
  H   -4.03026295088469     -4.22588674450545     -2.77051461986435
  H   -0.83334031584526     -1.34013367569061     -4.31681467118517
  H   -1.29077871076645     -2.65011261388673     -5.44581984000409
  H   1.09368058204918     -2.75678530355759     -5.04097681778695
  H   0.31795710551029     -4.19558374653888     -4.31406430698119
  H   2.95365901700578     -2.84242533441751     -3.46130771807587
  H   2.24570220659699     -4.34962396899231     -2.80796594623474
  H   3.75534754409540     -3.08498538650317     -1.21605056044287
  H   2.56151347810103     -1.74089862718769     -1.21379394613276
  H   -4.51525460444555     -4.48298000395886     -0.41699417356759
  H   -5.54351548418534     -3.01851899466733     -0.41136038050447
  H   -4.71543236589744     -3.36174616059458      1.82037592729456
  H   -3.90878363521883     -1.90819788803356      1.15180539580337
  H   -2.89852619326789     -3.84497737101610      3.33348421781711
  H   -2.11898827231822     -2.32745744973408      2.79434992528139
  H   3.32023482629338     -3.43775998928189      1.16375210530405
  H   2.14436805585934     -2.07967330936460      1.11685904728313
  H   1.77257850501794     -3.77052983143878      2.96492733676227
  H   1.40960846604455     -5.00344903378566      1.72003075153467
  H   -0.52601655812352     -4.00501350698771      3.75610668351370
  H   -0.94219741963235     -5.15004360560909      2.44607717902200
  K   -0.81453774344572     -2.49352991776427     -0.70411022533778
  F   0.65389971171556     -0.20756525908122     -0.13193372605632
  O   -1.76825745244710      0.17303971974587      0.32369697576866
  H   -0.76157475259505      0.08199936937079      0.17275821543902
  H   -1.87912437674879      0.23623572282045      1.27989391367109
```

KF-18C6-HF

```
46
Coordinates from ORCA-job geom
  C   -3.17648714660632    -2.52107220433726    -3.65063411548041
  O   -1.84866220524884    -2.98392592192982    -3.48594573938936
  C   -4.07569901485969    -3.18557951761542    -2.63631934710340
  C   -0.94581072651540    -2.46830582743708    -4.44752378758731
  C   0.42016805377426    -3.07793761793524    -4.24185719158481
  O   0.92064832040743    -2.70035604901708    -2.97480041038952
  O   -3.68585852405775    -2.80367937893540    -1.33192767162168
  C   2.23856620462255    -3.15350854499660    -2.72189188948105
  C   2.66058070014177    -2.75378322583083    -1.32796923934442
  C   -4.52253673043217    -3.32000098485773    -0.31327098237105
  C   -3.97891169716035    -2.93191640989944     1.04067765884257
  O   -2.75312158853246    -3.60391828911089     1.27339910973560
  O   1.88728466089666    -3.46052516921114    -0.37297727850413
  C   -2.21153917561749    -3.35009159330446     2.55801946957266
  C   2.26168905986828    -3.18673158601306     0.96645990538146
  C   1.36835869326992    -3.95517977007730     1.91206906258417
  C   -0.87871749860889    -4.04741233728975     2.69922036099306
  O   0.04705345040172    -3.46428939336363     1.80409410389930
  H   -3.54569648752761    -2.76401257836743    -4.66557545278439
  H   -3.21526624501798    -1.42069386032747    -3.53181281034363
  H   -5.12085002752985    -2.87841451709414    -2.83419879085637
  H   -4.02021614587279    -4.28625113921934    -2.74384093827689
  H   -0.88635553702761    -1.36572067125719    -4.36291663536931
  H   -1.29268869692891    -2.71004682796905    -5.47054917338623
  H   1.09214895071190    -2.72114991964010    -5.04620773274582
  H   0.36075839367324    -4.18110121729476    -4.31597114834062
  H   2.94232444722611    -2.70730516935290    -3.45097693665340
  H   2.29125075340277    -4.25391177427729    -2.83137133015007
  H   3.73455677993764    -2.99183418828623    -1.20249886689228
  H   2.52783638412050    -1.66480991054124    -1.18788541072298
  H   -4.58410397995481    -4.42245955543513    -0.39256625055868
  H   -5.54768492370304    -2.91509742360291    -0.41908495735259
  H   -4.72132955412549    -3.21009040858393     1.81338423041198
  H   -3.82233119342443    -1.83789451574055     1.08782601972254
  H   -2.89782963262114    -3.72182974257853     3.34353517688098
  H   -2.07877289582327    -2.26168270312124     2.70249995456248
  H   3.31287955795779    -3.48807176910155     1.14011264410570
  H   2.17415951174697    -2.10258179463334     1.16719883987540
  H   1.74510070528113    -3.82247005038176     2.94482513875997
  H   1.39930794158976    -5.03674966942152     1.67638212445202
  H   -0.52848531130929    -3.93538080157568     3.74366106404066
  H   -0.98610465991482    -5.13015536407666     2.49293334841224
  K   -0.86933449609397    -2.62035104768267    -0.73524073420142
  F   0.55165882391636    -0.13678471763233    -0.08236759139575
  F   -1.57207115332900    -0.26455055007073     0.73427342968978
  H   -0.50373614510335    -0.16891429157198     0.34753077096503
```

KOH

```
3
Coordinates from ORCA-job geom
```

```
  K   -8.94834345365432      1.58910814798209     -0.13504583783590
  O   -6.77886821046272      1.81637408456797      0.41627172275283
  H   -5.85000833588293      1.90959776744994      0.63959411508307
```

KOH-18C6

```
45
Coordinates from ORCA-job geom
  C   -3.24628445739868     -3.60796925176026     -4.77115422518336
  O   -1.85495655452978     -3.40621737113800     -4.61336828500917
  C   -3.77810808473502     -4.39191641282845     -3.59499941185484
  C   -1.23757113845311     -2.76762170645915     -5.71468643878236
  C   0.25127887867506     -2.67291979256717     -5.47694729141282
  O   0.50632034980045     -1.81420462934268     -4.38316866333045
  O   -3.66449614747268     -3.62040649764019     -2.41557869903562
  C   1.88062225432470     -1.66046910724157     -4.08530775843977
  C   2.04838698872821     -0.74008377614702     -2.89899599026488
  C   -4.11588779881677     -4.28980857115150     -1.25494774710604
  C   -3.94801087736317     -3.39530038641934     -0.05011912346709
  O   -2.57114168174834     -3.17344398150825      0.18866227650697
  O   1.52579591897103     -1.35754730041116     -1.73745185228106
  C   -2.30987523771897     -2.40600600884570      1.34859799230115
  C   1.66795827194142     -0.58038189594334     -0.56294421779687
  C   1.15223184357607     -1.35785658778481      0.62532028535843
  C   -0.81876980621420     -2.28812791867043      1.55959686666427
  O   -0.24500916164925     -1.54067566204210      0.50555884807770
  H   -3.45044320638493     -4.17714471448054     -5.69864702829973
  H   -3.76937167816365     -2.63498963817189     -4.84804629531890
  H   -4.83760058612489     -4.65165129143589     -3.78632276883456
  H   -3.21249203733771     -5.33792168933751     -3.48858816982520
  H   -1.66357468741930     -1.75598411548460     -5.86112534135205
  H   -1.41230338304844     -3.34584178507273     -6.64266436757995
  H   0.73544904051055     -2.28395827583047     -6.39390942295179
  H   0.66507698183575     -3.68039875983279     -5.27687201272914
  H   2.41644849360135     -1.22657604562207     -4.95196647057607
  H   2.33678209833905     -2.64506239673941     -3.86485220710574
  H   3.12634811057355     -0.52361529739360     -2.76715477031697
  H   1.53020893614799      0.21973717704065     -3.08889728890683
  H   -3.54541934171350     -5.22759071098364     -1.10847230909737
  H   -5.18598704901147     -4.55731630986098     -1.35462472988545
  H   -4.41627696221710     -3.88664627867826      0.82476883740477
  H   -4.46846825272077     -2.43215914600545     -0.21634966146479
  H   -2.75669646864824     -2.88986892424805      2.23875623049814
  H   -2.75908856490415     -1.39839337413662      1.25059967012546
  H   2.73352539667931     -0.33344759539640     -0.39061182221221
  H   1.11058838456390      0.37107984516907     -0.66443577798725
  H   1.38986254865460     -0.79768556577881      1.55059088946617
  H   1.66293965039380     -2.33857876491482      0.68319212878058
  H   -0.63575316590776     -1.78902960737421      2.53111529921475
  H   -0.36248087967337     -3.29597408268840      1.60632910630166
  K   -1.34788033506970     -1.82494754391087     -2.15370809771902
  O   -2.43870604242630      0.61132300296947     -2.35126942495880
  H   -2.59417056044552      0.89737874610005     -1.44027075961379
```

KOH-18C6-2H2O

```
51
```

```
Coordinates from ORCA-job geom
  C   -3.11078899910642     -2.37366605976673     -3.59238056263720
  O   -1.78972786344394     -2.85960068928163     -3.44107575898535
  C   -4.02530015438272     -3.08049031291386     -2.62064081111880
  C   -0.86916320573766     -2.30313353845017     -4.36197406552564
  C   0.47791507241318     -2.96600666142340     -4.19904734942279
  O   1.00800064616911     -2.66087592790455     -2.92403699151886
  O   -3.65666968958830     -2.75147158611612     -1.29673307120114
  C   2.27453346283078     -3.24214777224462     -2.67709976277743
  C   2.75091011164692     -2.85953581634002     -1.29587914387026
  C   -4.48303832924373     -3.34005205515791     -0.30925307905579
  C   -3.98652867540638     -2.96159414009156      1.06527214916498
  O   -2.72289700553209     -3.56593100877142      1.29548331126366
  O   1.90812104224703     -3.44205117146980     -0.31962623533947
  C   -2.20026783819775     -3.29579838821271      2.58485119693278
  C   2.30001630535459     -3.15514378260636      1.01320499112063
  C   1.39086725896025     -3.88298099576069      1.97539989181227
  C   -0.85618835484303     -3.96657067404416      2.74364249228910
  O   0.07991539139868     -3.36370010554759      1.87123001648688
  H   -3.47256607831435     -2.55918537589450     -4.62208414138840
  H   -3.13974721448639     -1.28097691144943     -3.41489751276158
  H   -5.06834006629201     -2.76750686649757     -2.82199622073194
  H   -3.96542466203643     -4.17568802868604     -2.77371963060118
  H   -0.77756684495929     -1.21137719136787     -4.19992809115711
  H   -1.21950649686254     -2.46189942501828     -5.39998200262983
  H   1.15437706662365     -2.60070282743894     -4.99607586330027
  H   0.37632160293019     -4.06201569388176     -4.31845265035306
  H   3.01329918212690     -2.88517878065790     -3.42069551506763
  H   2.21242202251290     -4.34406692751351     -2.76411617087973
  H   3.79176748177893     -3.21488810634665     -1.16740581598098
  H   2.75523031587997     -1.75741985496919     -1.19086018415645
  H   -4.48472799622467     -4.44154757532540     -0.42307982486541
  H   -5.52645037700880     -2.98682358453154     -0.42371439980083
  H   -4.72014043056170     -3.31753884622645      1.81497903690004
  H   -3.89732625526590     -1.86357133260419      1.15909301108150
  H   -2.88512565094341     -3.68349578630615      3.36422699221048
  H   -2.09928138219742     -2.20382529520381      2.72820918857815
  H   3.34128631514854     -3.48918939753474      1.18648803215582
  H   2.25089115340675     -2.06461350222046      1.19502738637414
  H   1.77873226502498     -3.74684294504709      3.00379519865485
  H   1.39251465633054     -4.96805133446965      1.75398290917123
  H   -0.52665694447087     -3.85839682700747      3.79539604977985
  H   -0.93980353912509     -5.04925881863859      2.52633038569846
  K   -0.84114921649213     -2.50707088208841     -0.69253340454141
  O   -0.72399739470118      0.10268271226574     -0.20650401598135
  O   -2.63052892494936      0.28084424512518      1.52758278158073
  H   -1.87577738422879      0.21676159192119      0.83094105306399
  H   -2.22862256130321      0.72117587333290      2.28622447779794
  H   -0.90400405459185      0.68483952091063     -0.95548036326648
  H   2.23677024545767      0.48121503925550      0.04521208808163
  H   0.68827487290317      0.39119424944904      0.36545385550438
  O   1.61371711935264      0.58792957076896      0.77377614321334
```

KOH-18C6-H2O

```
48
Coordinates from ORCA-job geom
```

```
  C   -3.16127786339773     -2.48369986465242     -3.65535581432034
  O   -1.85444076101116     -3.00176933003878     -3.49596315237169
  C   -4.08662889630283     -3.12644144673307     -2.65053024371916
  C   -0.93539188607004     -2.54106580901787     -4.46835411411068
  C   0.40827694213207     -3.19430469905073     -4.24610279988566
  O   0.92872699707243     -2.78983481205183     -2.99657781452854
  O   -3.69147712751302     -2.77324988399307     -1.34072153599974
  C   2.23734350440616     -3.26157454306892     -2.73591884921159
  C   2.67346375614437     -2.80966261001785     -1.36245708041129
  C   -4.51956992911076     -3.32578734585394     -0.33569188934007
  C   -3.98875672715471     -2.96401440173441      1.03056704192758
  O   -2.74636503700879     -3.60948801007112      1.24418818166046
  O   1.91970529530737     -3.49192831564386     -0.37555421909357
  C   -2.23174588761562     -3.43044344768668      2.54942545481392
  C   2.24331402636818     -3.09792789051063      0.94685780560951
  C   1.37668454112990     -3.85439139302542      1.92586999622699
  C   -0.87290509293179     -4.08190723741066      2.65741011284939
  O   0.03858646959313     -3.40905090275714      1.81298210439346
  H   -3.54011584892513     -2.69796466922037     -4.67344540263405
  H   -3.15716368397391     -1.38409171833002     -3.52179737932407
  H   -5.12100966347660     -2.78361067487236     -2.84928251454833
  H   -4.06701996138327     -4.22709903656546     -2.77081753566395
  H   -0.83506394396219     -1.43960480739420     -4.40950587942580
  H   -1.29195994202147     -2.79297627241559     -5.48598947203890
  H   1.08868011100100     -2.89294969767108     -5.06634930574245
  H   0.30556707842466     -4.29649446021992     -4.28112476374255
  H   2.94407442648755     -2.86130090958463     -3.48889623938141
  H   2.26645036790663     -4.36679571536715     -2.80029631375217
  H   3.75234876217261     -3.02942288052177     -1.24012525506807
  H   2.51861732063604     -1.71969848746270     -1.25873236209389
  H   -4.56236865950601     -4.42717511526505     -0.44151547369962
  H   -5.55216712571493     -2.93688082822299     -0.43326180545366
  H   -4.72565279707100     -3.28810340266504      1.79089330123457
  H   -3.86346009043390     -1.86791889372589      1.11345091503855
  H   -2.91009838714583     -3.88649113049173      3.29701828456182
  H   -2.14783605556141     -2.35181490303381      2.78277528457541
  H   3.30650739336432     -3.32201321845623      1.16292634387723
  H   2.08102813879665     -2.00988290019712      1.06086449720393
  H   1.75277885906578     -3.67736348848250      2.95243789549525
  H   1.43827177224592     -4.94198848335725      1.72607057808104
  H   -0.53703626006506     -4.02773317796146      3.71132546145190
  H   -0.93782621756177     -5.15189124942428      2.37869581585068
  K   -0.81475893076417     -2.43926710768807     -0.73492848842507
  O   0.55184698453743     -0.12413666611025     -0.20615688372694
  O   -1.74185084204345      0.07297202816476      0.77525491225607
  H   -0.75532546463539      0.05473748647834      0.36456520446618
  H   -1.62010340244145     -0.03340330500322      1.72647351893433
  H   0.68211373801127      0.44181562838567     -0.97752012279497
```

PE2

```
20
Coordinates from ORCA-job geom
  C   0.67887775616314      1.35218721843516     -0.11517514845532
  C   2.01383263526751      0.95943610846455     -0.17185694713807
  C   2.36124690917309     -0.39646377313845     -0.09734047281748
  C   1.35192538717161     -1.35049274508159      0.03437142793139
```

```
  C   0.00394621918315     -0.97240163461247      0.09217204713189
  C   -0.33446457620987     0.38689345658403      0.01790249875170
  H   0.39605943769244      2.40576463935947     -0.17136289484071
  H   2.79229691741685      1.72005543110380     -0.27537465090852
  H   3.40901981766998     -0.70214100991417     -0.14198674776762
  H   1.60643202997840     -2.41211309575289      0.09315859113049
  H   -0.76349721040000    -1.73972804230139      0.19288427516899
  O   -1.61161075576415     0.86186435776397      0.06207315321035
  C   -2.69493704209806    -0.05317062355569      0.23202162058012
  C   -3.96923650758714     0.72881613995094      0.32279913687829
  H   -2.53598545258677    -0.63110299007520      1.16248688042212
  H   -2.72975514475673    -0.76676711417078     -0.61003205551101
  C   -5.03907488343761     0.51029425708789     -0.44505347086482
  H   -5.96049338666625     1.08461910427368     -0.31396305748517
  H   -5.03650802032130    -0.25682007375923     -1.22690108140345
  H   -3.99446412988828     1.50405038933837      1.09828689598685
```

RBr

```
22
Coordinates from ORCA-job geom
  C   0.76297374601399      1.36686942278259      0.01090595688680
  C   2.07127833551952      0.88895176599985      0.01228418622881
  C   2.32663498669857     -0.48888614406267     -0.01344963636229
  C   1.25270340363766     -1.37912033554714     -0.04080419932921
  C   -0.06930337465136    -0.91506763109750     -0.04246318881276
  C   -0.31495104556902     0.46553479098814     -0.01641845788331
  H   0.55160397164953      2.43827805603986      0.03089827670485
  H   2.90150706155019      1.59990738324354      0.03385715904361
  H   3.35364768160158     -0.86100099145239     -0.01221600134801
  H   1.43504613113330     -2.45686400225384     -0.06118912398879
  H   -0.88959173433393    -1.63246616285844     -0.06395109503569
  O   -1.56043724887470     1.02185405546291     -0.01501999116397
  C   -2.70536303099558     0.18132260173868     -0.03991185667599
  C   -3.93703491292677     1.08148316529945     -0.03067506999265
  H   -2.70270912490089    -0.48774096192709      0.83984887268202
  H   -2.69043724564416    -0.45212421669547     -0.94551319579574
  C   -5.21204311852622     0.25674531491258     -0.05004299374713
  H   -3.90179871389638     1.74695559644162     -0.90782911392679
  H   -3.90990366666750     1.71726674928420      0.86852897150277
  Br  -6.82491126127161     1.40537832954973     -0.04788431692874
  H   -5.31762837532236    -0.38030711047138      0.83688005577378
  H   -5.30452246422387    -0.35876967537722     -0.95352523783157
```

RF

```
22
Coordinates from ORCA-job geom
  C   0.77545089843817      1.36710856977718      0.01081445390800
  C   2.08989243669190      0.90671005089929      0.01147503120535
  C   2.36374000702960     -0.46771901242507     -0.01419778620858
  C   1.30170674533396     -1.37200503551430     -0.04071260904221
  C   -0.02649960377126    -0.92581478257355     -0.04166515787868
  C   -0.29033226108857     0.45144679637724     -0.01576759640687
  H   0.55022895501712      2.43569799465244      0.03069637648737
```

```
  H   2.91045044989383      1.62883780034958      0.03238748300136
  H   3.39553833227440     -0.82627954630008     -0.01359824136362
  H   1.49851613128732     -2.44722113520862     -0.06105039623050
  H   -0.83666278934110     -1.65461180155571     -0.06258879676704
  O   -1.54293029378535      0.99077328108208     -0.01419960846858
  C   -2.67614735282252      0.13419955589364     -0.03807681036185
  C   -3.91927795701002      1.01196215413438     -0.02985519979834
  H   -2.66136468644716     -0.53456228477592      0.84193111186679
  H   -2.64966053738668     -0.50063690761284     -0.94258517837328
  C   -5.19064781073655      0.18621260783462     -0.05195824241449
  H   -3.90069950940047      1.67780782348208     -0.90807006754726
  H   -3.91115112709951      1.64648232547663      0.87141812338781
  F   -6.30627795737129      1.04384354246125     -0.04641128281949
  H   -5.28053995811413     -0.46181098556648      0.83422924035494
  H   -5.26784211159169     -0.43340101088785     -0.95942484653083
```

TS1-18C6

```
66
Coordinates from ORCA-job geom
  C   -3.23752588664845     -3.61245757254523     -4.78082172666006
  O   -1.85323744708540     -3.38096315705574     -4.59361502141944
  C   -3.78946422608544     -4.35847121684183     -3.58996282223796
  C   -1.19911049795499     -2.84214572198434     -5.72751153535485
  C   0.27956386294376     -2.71569389344587     -5.44728009443794
  O   0.49283340947848     -1.78557074502322     -4.40140942385524
  O   -3.67925023751118     -3.56153865309965     -2.42495269226833
  C   1.86196398772091     -1.57309820995523     -4.10488571962704
  C   1.99304066218057     -0.65421311306771     -2.91333503219198
  C   -4.17424997964453     -4.20232240993889     -1.26347225824666
  C   -3.97013642165178     -3.31769364146616     -0.05745404382289
  O   -2.58421357620650     -3.15180093980794      0.18013494227437
  O   1.48278177623734     -1.29814336234725     -1.75999917642401
  C   -2.28958306224356     -2.43143507014392      1.36335000934924
  C   1.65528709926858     -0.56090168502574     -0.56328354617090
  C   1.16430506011777     -1.37980973373066      0.60742740993794
  C   -0.79308557584364     -2.34066734775327      1.54611618383789
  O   -0.23319150009350     -1.57236813640665      0.49848603793434
  H   -3.40673109242898     -4.22342014863691     -5.68793447672057
  H   -3.77352940902502     -2.65279620658317     -4.91660437907629
  H   -4.84945255911921     -4.61017998036284     -3.78696405545935
  H   -3.23630778682624     -5.30752600419136     -3.45242981105627
  H   -1.62542546950263     -1.85312531793143     -5.98478330726277
  H   -1.33802402460266     -3.50472980208167     -6.60322970405068
  H   0.78908096943236     -2.38180256950283     -6.37174058650654
  H   0.69773818342574     -3.70221880477318     -5.16889810025271
  H   2.37512167983658     -1.11347401141786     -4.97139154481559
  H   2.35748191886595     -2.53877933114455     -3.88839829852286
  H   3.06291513300408     -0.40659796449016     -2.77422369077799
  H   1.44850048434096      0.29212332100656     -3.09838445840678
  H   -3.65305036389731     -5.16718472326517     -1.11170532252726
  H   -5.25521762266374     -4.41532647087435     -1.37247042605245
  H   -4.45626133624751     -3.79445902582248      0.81529878059638
  H   -4.45254171269730     -2.33361471254265     -0.21640081869342
  H   -2.72586033045359     -2.94284261755826      2.24292833917653
  H   -2.72631188648593     -1.41535416198662      1.31077683993053
  H   2.72535704072826     -0.32376510490750     -0.40797944783501
```

```
H   1.10016173900179      0.39554912274923     -0.61848331147907
H   1.40783075065094     -0.84549467681287      1.54601281036348
H   1.68446629599109     -2.35687974999376      0.62852196133849
H   -0.58312957966895     -1.86895618957372      2.52548109497929
H   -0.34832160962309     -3.35457971092057      1.55649296259161
K   -1.32764110447839     -1.89345009204899     -2.12676156883382
F   -2.34026841176189      0.51550386654224     -2.32879150039684
H   2.45193882685560      0.04155071532634     -8.46890018686587
H   -1.58279727360762      1.20287534995118     -6.41966886548545
H   -3.08727839252966     -0.01498878391345     -4.33019843998562
C   2.26464478463825      0.99366895161236     -7.96506296355101
H   0.45891632451570      0.26876802329972     -7.03189363724654
C   1.13179915200082      1.11789619175303     -7.14937961237905
C   3.14505222979012      2.06192818427646     -8.14003003196231
H   4.02505922771787      1.95515045962559     -8.77838730952997
C   -1.13397671938662      1.51806315101100     -5.46133571624764
C   -3.23742365573766      1.04310248763918     -4.15603300361407
H   -2.67881421701757      2.94723613533674     -4.96337491379349
C   0.87961141597032      2.33514061216971     -6.49804717280162
C   2.88533064234449      3.27424466998248     -7.48636623325492
C   -2.20156139778286      2.05754103133139     -4.52571814228998
O   -0.18968234586801      2.56026831812877     -5.68624322587566
Br  -4.70747350064587      1.08462952967009     -6.11100050286182
C   1.76380791909389      3.41414494542200     -6.67238783276759
H   -0.63221506089282      0.64436124476153     -5.00901994077852
H   -4.07019832081277      1.33252902736194     -3.52685699219627
H   3.56435334465681      4.12162419430515     -7.61329689444509
H   1.55166794876261      4.35442985041001     -6.15858390415711
H   -1.73127827483847      2.37313138730319     -3.58108394877595
```

TS1-18C6-H2O

```
69
Coordinates from ORCA-job geom
C   -2.98445157461378     -3.64201756079372     -4.56816989180280
O   -1.64389929066273     -3.22764207560117     -4.37486663220404
C   -3.44194638313660     -4.44807431598940     -3.37663692154465
C   -1.08361161284060     -2.57115319745567     -5.49714321530827
C   0.36891288821376     -2.25470658390019     -5.23305217538878
O   0.47590569129022     -1.34199865757045     -4.15628220481785
O   -3.46097439846758     -3.62831444503845     -2.22323293985625
C   1.80881905876404     -0.95036836797283     -3.88127992116151
C   1.83550153071183     -0.03832305951964     -2.67870056794134
C   -3.88193432273779     -4.30668465714119     -1.05363287673572
C   -3.81794571876349     -3.37114428767735      0.12965776576765
O   -2.47023704034409     -3.01768471113968      0.37781193824777
O   1.41424703030882     -0.75570025995788     -1.53302306255704
C   -2.29291326760778     -2.21508918078067      1.53575958585077
C   1.50253506096404     -0.01576016335734     -0.32916595455042
C   1.07330061806507     -0.88367486778902      0.83011850961740
C   -0.81867525596025     -1.97708650945993      1.76111771818315
O   -0.29679516678217     -1.20603402543373      0.69526635497794
H   -3.06503887298205     -4.27212932885853     -5.47440573025146
H   -3.64154829971478     -2.76193177591087     -4.70972912017709
H   -4.45345481366434     -4.84807896667385     -3.58290847052116
H   -2.76257384545020     -5.30796805438250     -3.22035391357596
H   -1.64387728215518     -1.64135561258767     -5.71630758052329
```

```
  H   -1.14697043286652     -3.21873891798000     -6.39267624617126
  H   0.80822329673664     -1.82002695768514     -6.15121543356617
  H   0.92226848852769     -3.18443413894420     -4.99826511602753
  H   2.23790252959873     -0.41381509056305     -4.74917043964743
  H   2.43392597386939     -1.84353925619847     -3.68971161084395
  H   2.86789988506024      0.33672126769019     -2.54133403423857
  H   1.17776396690712      0.83547774318025     -2.84786354876760
  H   -3.23472769083780     -5.18615953768713     -0.87191534121833
  H   -4.92154956515185     -4.66860799133296     -1.17151723087659
  H   -4.25068322603480     -3.88417101438238      1.01017737268629
  H   -4.42285406725283     -2.46444988920595     -0.06426386946819
  H   -2.70859169496748     -2.73015571575342      2.42316733998586
  H   -2.81959951249058     -1.25093384562225      1.40867901877891
  H   2.54377427282842      0.31956407554096     -0.15891747762859
  H   0.85976568002033      0.88368859074185     -0.38597337167895
  H   1.24856902136865     -0.33091804096054      1.77334025233698
  H   1.68437739126064     -1.80668912013605      0.86050812148415
  H   -0.68742391982648     -1.44316937037554      2.72223777840833
  H   -0.28032559138503     -2.94202487838190      1.83414753308617
  K   -1.33624533638059     -1.67034727028336     -1.92013785892671
  F   -2.33619000563083      0.74671286547108     -2.01366298942859
  H   0.65894123688399      0.08923712735758     -9.19620144140632
  H   -1.83912355724039      2.17518332574785     -5.88768237764018
  H   -3.48903608754961      1.24724940141595     -3.80058764588817
  C   1.06813119566409      0.79346410730672     -8.46692521164660
  H   -0.69988358164679      0.70997265257380     -7.23270795332959
  C   0.29282233480075      1.14335009312481     -7.35322374848166
  C   2.34286892156687      1.32972750764984     -8.65276728798345
  H   2.93901731985577      1.05058779592727     -9.52447651710042
  C   -1.16907393649442      1.94835207436747     -5.04064584262571
  C   -3.02617651969359      2.11686714134592     -3.34933902682874
  H   -1.69816219934840      3.70370099218434     -3.89095037085064
  C   0.80634501827125      2.04771283339791     -6.41105919749116
  C   2.84810849311442      2.23212484159530     -7.70688098629880
  C   -1.68045473644675      2.61025825587284     -3.77301084540290
  O   0.13567315478720      2.45489267668853     -5.29826131045228
  Br  -4.52485741220482      3.47478780844256     -4.84401532588801
  C   2.09014701934085      2.59069128350433     -6.59436087044917
  H   -1.12415483801335      0.85302101255829     -4.90977610862388
  H   -3.52840098493486      2.58139297382009     -2.50891681825806
  H   3.84421172947004      2.66314068999558     -7.83792180006221
  H   2.47279316110368      3.29460702696763     -5.85203360135826
  H   -0.99428543066180      2.38926827273613     -2.93962466264040
  O   -3.81446137910147      0.73278858389028      0.13761313762063
  H   -3.24144174929170      0.73265298098875     -0.67686044873336
  H   -4.69673136801817      0.50272569840008     -0.17945728018536
```

TS1-H2O

```
25
Coordinates from ORCA-job geom
  H   0.89477205300517      0.52599612801213     -8.48836788523959
  H   -2.73025306294958      1.18099549056962     -5.60672313457577
  H   -3.62856551824406      0.32835060036051     -3.23854881895750
  C   0.49566981196393      1.50400100832924     -8.20666956651888
  H   -0.67446999282849      0.68082808288778     -6.59228192171695
  C   -0.39542435709643      1.58771078571780     -7.12874676770528
```

```
  C   0.87369388845386      2.64433057920707     -8.91615596688320
  H   1.56890149488285      2.56836627676967     -9.75531819202966
  C   -2.21746779690953      1.91612634739307     -4.96169399273832
  C   -3.77126934777439      1.38677234653949     -3.03409501807418
  H   -4.01835894041589      2.96552571841519     -4.39851594650102
  C   -0.91186938435521      2.83755113506988     -6.75896820622383
  C   0.35089275848378      3.88885886439601     -8.53920887457637
  C   -3.18200726428940      2.44325241975238     -3.90950104961869
  O   -1.78326273616441      3.02876712261553     -5.72497288467503
  Br  -5.95017400625898      0.70204670509671     -4.66422543948846
  C   -0.53546385690667      3.98963014674392     -7.46941328396345
  H   -1.35078658514365      1.41504547347302     -4.49276703288170
  H   -4.59277493266312      1.64363166629112     -2.36978493762748
  H   0.63636135455749      4.79168902411202     -9.08549177258110
  H   -0.94999537794872      4.95369093772050     -7.16644488486833
  H   -2.68910417796841      3.20620562975127     -3.28266510430996
  O   -2.56611337040355      1.26874714568385     -1.54242021472774
  H   -1.66572792966332      0.96886742227831     -1.75984310851188
  H   -2.46615272336323      2.10628294281385     -1.05545599500552
```

TS2-18C6

```
66
Coordinates from ORCA-job geom
  C   -2.86290364009140     -3.08679622123610     -5.51596425274931
  O   -1.71834973938635     -3.57835407938059     -4.83771818424698
  C   -4.06759615519077     -3.18278506627917     -4.61067275561400
  C   -0.54302811696029     -3.55754480602705     -5.62838073273536
  C   0.61131093573403     -4.12561673898305     -4.83756057539638
  O   0.92588065285728     -3.25499551983000     -3.76657041089401
  O   -3.92154166326156     -2.27940770114676     -3.52896672637449
  C   1.98006411423415     -3.71410674618594     -2.94289176481086
  C   2.28654329955233     -2.67406506600952     -1.89209806772145
  C   -4.97396754264179     -2.33985422235948     -2.58619110787356
  C   -4.76864375521320     -1.27720348812003     -1.53296533229858
  O   -3.57576582868959     -1.54171199056014     -0.81760569656666
  O   1.16668488745470     -2.51428562047849     -1.03965678469377
  C   -3.31434587757219     -0.61695103184348      0.22210712658039
  C   1.37288132066000     -1.57116243139741     -0.00372004890833
  C   0.18003267606315     -1.56204778917327      0.92057353168552
  C   -2.13239324023301     -1.09011894925039      1.03234264301192
  O   -0.96885614021308     -1.11162026964940      0.22515411917525
  H   -3.05338385725908     -3.68564405309374     -6.42723997870329
  H   -2.70210803422843     -2.03599700975132     -5.82022906374435
  H   -4.97591833654143     -2.93738081787041     -5.19358039001952
  H   -4.17567549571660     -4.21710953403330     -4.23091885036791
  H   -0.31369219732010     -2.52068010271424     -5.93898678178002
  H   -0.68463262177933     -4.16839333494853     -6.54074372210061
  H   1.48529259354830     -4.23971437701454     -5.50741371931376
  H   0.34785644102365     -5.12862781633858     -4.44947922518581
  H   2.89265886356476     -3.89061046528490     -3.54417614521065
  H   1.70107604630996     -4.67170249835630     -2.46211447076463
  H   3.16920875821756     -3.00153350389788     -1.30992059083159
  H   2.53833323129509     -1.71058027845029     -2.37588862342338
  H   -5.00808242551106     -3.34127213592684     -2.11476234178735
  H   -5.94880680195264     -2.16574204420696     -3.08114090653043
  H   -5.63753871076386     -1.27850139880757     -0.84728187347737
```

```
  H   -4.71626720080363     -0.27829334216234     -2.00758894396808
  H   -4.19028138456533     -0.53324642269745      0.89325764346169
  H   -3.11557088388699      0.38821931797450     -0.19876957479196
  H   2.27222388418781     -1.83512054399337      0.58514418185299
  H   1.53533643226264     -0.56234445383882     -0.43108759644141
  H   0.39701455509157     -0.89469928827940      1.77681830433689
  H   0.00543486477732     -2.57950828393441      1.31995355977450
  H   -1.99215611042864     -0.40722901709434      1.89222633231761
  H   -2.33374881079500     -2.10231462411886      1.43254274602725
  K   -1.25921561836783     -1.90253669353932     -2.56183785596527
  F   -0.96159647914187     -0.01730132386933     -4.42202166826780
  H   -7.97277707094564     -0.91950925499848     -7.28861692144218
  H   -4.74633879853215      1.94287505820270     -5.57790976293123
  H   -3.30064549907298      1.81830700506258     -2.74272488391704
  C   -6.92999637602453     -0.89352718533585     -7.61610463440195
  H   -6.31676882514613      0.29478672135624     -5.92358552015326
  C   -5.99241411945552     -0.20151270183469     -6.83782181540658
  C   -6.55110349584083     -1.54181268337619     -8.79197983079638
  H   -7.29007763959297     -2.07885909769622     -9.39087378666955
  C   -3.97706360762582      1.17660622158905     -5.36210898051949
  C   -2.76662561497201      2.36660591205823     -3.52297774573229
  H   -2.21212660669344      2.45298155696495     -5.57322752739161
  C   -4.65033125793973     -0.15799893479462     -7.24782614412171
  C   -5.20920537621781     -1.49490531445807     -9.19441652829100
  C   -2.72099747879413      1.82287362359251     -4.82801753162403
  O   -3.66170024634785      0.47798050270328     -6.56506185647181
  Br  -4.25526954614927      4.26141710912300     -3.28129056828480
  C   -4.26499351175977     -0.80983826089342     -8.43337221741764
  H   -4.39940257607899      0.47393683422683     -4.62083825724033
  H   -1.90399111373865      2.93305496902807     -3.16871779366397
  H   -4.89442835782429     -1.99780684101973    -10.11266940081813
  H   -3.21685347866665     -0.76670516620482     -8.73769159755834
  H   -1.82092629089870      0.87195171086431     -4.57918211981049
```

TS2-18C6-H2O

```
69
Coordinates from ORCA-job geom
  C   -4.61222599101399     -3.13300455395954     -4.02454738855183
  O   -3.31501862302737     -3.69968154831874     -3.98283251327010
  C   -5.04061970864353     -2.72862361456442     -2.63391481987816
  C   -2.84846431180350     -4.15554308440971     -5.24111740086236
  C   -1.50593394149880     -4.82739195761347     -5.07394284216333
  O   -0.54881700952249     -3.88508797069586     -4.62680151953001
  O   -4.19392188577809     -1.70222802879019     -2.14803978605910
  C   0.75826509752056     -4.41686236291275     -4.49811330042615
  C   1.70550445769676     -3.33713407407365     -4.03169479209214
  C   -4.55627879787360     -1.22805596800768     -0.86336616091407
  C   -3.57470461126348     -0.17329403442676     -0.41111703892203
  O   -2.29488360753497     -0.75537850063007     -0.23931471256306
  O   1.33985396706574     -2.92385811817799     -2.72447194651614
  C   -1.32186384670651      0.14399067761831      0.26262740674765
  C   2.20657121764931     -1.95005749059470     -2.16892809789346
  C   1.75630092211319     -1.61230600297476     -0.76671203134134
  C   -0.00814322985250     -0.57866716560942      0.44699100128754
  O   0.48239358773723     -0.99606481840699     -0.81293745301776
  H   -5.34037831778769     -3.86651380358767     -4.42034137455047
```

```
  H   -4.62376997385296     -2.25270994243348     -4.69599287369756
  H   -6.08898611725215     -2.37586304992771     -2.67534395312282
  H   -5.00446942163225     -3.60321018957181     -1.95601794907207
  H   -2.76720074894931     -3.30724678434268     -5.94788012131936
  H   -3.55872484251462     -4.88704238180827     -5.67153771165937
  H   -1.20046103836231     -5.25452472068023     -6.04844835535647
  H   -1.58801105712447     -5.66106789981860     -4.34984895075783
  H   1.11120474755753     -4.80615807368002     -5.47246294223904
  H   0.75602952793125     -5.25815559168294     -3.77826159919051
  H   2.73371133830099     -3.74778585337253     -4.02886762330774
  H   1.67914409703027     -2.47351652630314     -4.72188134126976
  H   -4.56953572273292     -2.06516461700037     -0.13914226642274
  H   -5.57072805719612     -0.78580117569952     -0.88914686324710
  H   -3.93307089166841      0.25813655686002      0.54303662466997
  H   -3.52458230068028      0.64609771767222     -1.15412358559478
  H   -1.64494440569678      0.55360558888553      1.23881971358822
  H   -1.19171691685846      0.99259331338334     -0.43619495917976
  H   3.24227907979311     -2.33881311787369     -2.12421124428141
  H   2.21025497561093     -1.04112928885503     -2.79961260099458
  H   2.49729442253567     -0.92825245366728     -0.31015309149462
  H   1.71507645750132     -2.52967378179706     -0.14808019552467
  H   0.71011466395799      0.11042456123930      0.93136653638564
  H   -0.14329836124693     -1.45184810138460      1.11419459846123
  K   -1.39515451525011     -2.03289335055956     -2.65347567686011
  F   -1.24204181015519      0.15282481951095     -4.11848094868055
  H   -4.89355865477380     -2.38197914326758    -10.30688978147416
  H   -4.28266520134797      1.01503603411437     -7.19761137966305
  H   -4.54554643074861      0.72428745329831     -3.97759635226467
  C   -3.91839088155490     -2.00570946012824     -9.98608813347867
  H   -4.76244880471239     -0.93814048550799     -8.31328053400000
  C   -3.84823000562605     -1.18531243761430     -8.85237777176684
  C   -2.76946575804145     -2.34265057990512    -10.70227787906023
  H   -2.83516729935352     -2.98329928584663    -11.58468923999093
  C   -3.55090656616391      0.48282951387530     -6.56004125608635
  C   -3.94001317084219      1.56078340023482     -4.33362717987187
  H   -2.54282524146006      2.26832869948448     -5.77296873680243
  C   -2.60253628830883     -0.69468229742287     -8.43060403013661
  C   -1.52927675601759     -1.84907204043233    -10.27432809598067
  C   -3.09283115521845      1.37804389466292     -5.43517647194224
  O   -2.42373760305182      0.10657317720649     -7.34480886820754
  Br  -6.05649981200636      2.82907684137508     -4.86607357591943
  C   -1.44191186012143     -1.03187339123185     -9.14983158995491
  H   -4.05351623612452     -0.41786467716205     -6.16175762105064
  H   -3.67480431255723      2.30220122630807     -3.57882535059775
  H   -0.61912309009632     -2.10357264267287    -10.82394892608866
  H   -0.48096017526964     -0.64091041829560     -8.80769276479075
  H   -2.15322907577077      0.72469974564395     -4.71093655003563
  O   1.18327719078313     -0.01296760914667     -5.26993281386257
  H   1.16708580898223      0.62072230690613     -5.99822651692955
  H   0.28936288288065      0.05212893856758     -4.86098642935928
```

TS3-18C6

```
67
Coordinates from ORCA-job geom
  C   -3.40422259023924     -3.96134219342750     -4.57614407981400
  O   -2.02241406067815     -3.64944970691497     -4.58839355752436
```

C	-3.76045486013808	-4.65756696093160	-3.28301433993831
C	-1.58404608954156	-3.03959868403633	-5.78809129996912
C	-0.08847126829889	-2.83438608714593	-5.73765004003305
O	0.24019379948820	-1.91018863564910	-4.71636823622431
O	-3.60820047912614	-3.76059776524301	-2.19994397948601
C	1.63463655589068	-1.70902508382623	-4.56699570407201
C	1.89792426629136	-0.70557855138274	-3.46975045479294
C	-3.93591652935801	-4.31374360797576	-0.93993107973567
C	-3.73771861491762	-3.27336969603703	0.13770341903770
O	-2.36120940720439	-2.96262233907906	0.25242876167622
O	1.47937295695865	-1.23369155327779	-2.22415389520253
C	-2.07353595678536	-1.99210014681953	1.24179452453723
C	1.74927880353533	-0.37993213658098	-1.12771685458766
C	1.32496309907904	-1.05120738076077	0.15646680730765
C	-0.57850805800500	-1.80581687720230	1.34960473520512
O	-0.07655507121268	-1.24154225616689	0.15262905964105
H	-3.65319787699036	-4.63188781885539	-5.42110122700298
H	-4.00547598620180	-3.03825257592684	-4.68593636954894
H	-4.80796262107523	-5.01101261551211	-3.34644520430819
H	-3.11246001954988	-5.54401680767550	-3.14042491295336
H	-2.09874450642841	-2.07056476903125	-5.93726325672174
H	-1.82229792376654	-3.68281741278652	-6.65702833408863
H	0.25400724983594	-2.45790524499761	-6.72089652739463
H	0.41646860637964	-3.80147090899895	-5.55053690997876
H	2.07016302790348	-1.32445941110693	-5.50874270934518
H	2.13174345774270	-2.66878369069185	-4.32774261509067
H	2.98215150551104	-0.48335213443088	-3.44805336691225
H	1.36630985056139	0.24166666718382	-3.68068099973528
H	-3.30219817217366	-5.19772169656560	-0.73254090548303
H	-4.99248952903552	-4.64489856277823	-0.92737725943741
H	-4.12281987667785	-3.67343284730288	1.09566222078668
H	-4.31624319009245	-2.36157348236939	-0.10668471286023
H	-2.46171189293995	-2.31829677730643	2.22602528110436
H	-2.55954504246255	-1.03075871924025	0.98564685019647
H	2.83183057410861	-0.15504444386003	-1.07122501759956
H	1.21183735833535	0.58071561604475	-1.24927826795713
H	1.62558825080590	-0.41029490812330	1.00782587953337
H	1.84221697271863	-2.02401804129090	0.26585788249332
H	-0.36256712752610	-1.14050580904738	2.20795134359860
H	-0.09108828022423	-2.78027555689173	1.54587848245848
K	-1.36983452266080	-1.93218947360671	-2.31229507464485
O	-3.23054210968224	-0.02542235552379	-2.60529110394286
H	2.54241969937346	0.76233126955078	-8.54711663045234
H	-1.45840371691034	1.77926008571322	-6.45562157944955
H	-3.49133998007613	0.07607495096232	-4.94804824763738
C	2.45001828739883	1.40047546432603	-7.66414963578014
H	0.41950605035304	0.77236036222179	-7.28770016840014
C	1.24194784841585	1.40460758440427	-6.95353591491651
C	3.52464316747268	2.19401355259584	-7.26090882167910
H	4.46212413642449	2.18286663720226	-7.82148647089889
C	-1.16699411334631	1.53809620310597	-5.41984698919392
C	-3.54237461078738	1.11336144809885	-4.63679882684301
H	-2.48914106383202	2.98023057701918	-4.50636059926673
C	1.11095951655659	2.22055215622596	-5.81889699115249
C	3.38584669584213	3.00523870894393	-6.12626443854642
C	-2.27958357001768	1.90049531441553	-4.45005487840425
O	-0.01269334133548	2.29401992666197	-5.05495602903852
Br	-4.41129278615350	1.85827149407473	-6.66711169701196

```
  C   2.19161603841827      3.02206271526369     -5.40958889941616
  H   -0.93808317584278      0.45872062245010      -5.36414403566773
  H   -4.41916321957679      1.40931844439415      -4.07522922116629
  H   4.21755188693489      3.63348660172324     -5.79634638198846
  H   2.07216792695171      3.65047721724812     -4.52411500795567
  H   -1.95198526780277      1.67724741615589      -3.42210034872124
  H   -4.01352108061383     -0.58416530960822      -2.49563513760304
```

TS3-18C6-H2O

```
70
Coordinates from ORCA-job geom
  C   -3.34650178242318     -3.90292093434772      -4.60963667156699
  O   -1.95961379520597     -3.61381156820819      -4.59263765933007
  C   -3.74737822249443     -4.57175987307449      -3.31520896936825
  C   -1.49341724170938     -2.98698680679343      -5.77333949612933
  C   0.00500171205448     -2.81191491709235     -5.70071638489806
  O   0.33911817625560     -1.91827874623997     -4.65382716786717
  O   -3.59913294783174     -3.66098933595744      -2.24219892174510
  C   1.73480746838092     -1.72614373480317     -4.50153796353486
  C   2.00309123814717     -0.73767273033365     -3.39184949512111
  C   -3.97806340854779     -4.18340534924879      -0.98191938208034
  C   -3.76785971121465     -3.13631596299418       0.08703608069590
  O   -2.38384131944221     -2.87967609607136       0.24228636420435
  O   1.56790281102013     -1.27143400193169     -2.15377182555294
  C   -2.09618192109112     -1.89026575572165       1.21822604813751
  C   1.84580226202699     -0.43252873587168     -1.04696874025860
  C   1.36679974898166     -1.09035667714263      0.22524170116083
  C   -0.60013098497982     -1.75591105562214      1.37517740632418
  O   -0.04022263223527     -1.22912286649590      0.18589531315366
  H   -3.58635313355575     -4.58166446511832      -5.45063513944025
  H   -3.92957291746852     -2.97213674776574      -4.74923507896244
  H   -4.80082551951545     -4.90239054511897      -3.39748275028877
  H   -3.12337517297222     -5.47005927894675      -3.14339025922234
  H   -1.98796472740695     -2.00517634280096      -5.90600618112870
  H   -1.73279427207498     -3.60652397601171      -6.65889610740287
  H   0.36507752291964     -2.41790840831658     -6.67053170482811
  H   0.49031581431386     -3.79231768815187     -5.53170805803513
  H   2.17177503965330     -1.33075735808797     -5.43812350760598
  H   2.22707952084282     -2.69168188837244     -4.27588002559449
  H   3.08992809221710     -0.53052734206399     -3.36029936595814
  H   1.48559378398228      0.21854547714889     -3.59800321625241
  H   -3.38347226743834     -5.08763363853753      -0.74791801901829
  H   -5.04620715934921     -4.47442700506142      -0.99238760292656
  H   -4.19837284171403     -3.50693848742450       1.03754283622431
  H   -4.30008424656801     -2.20540672264995      -0.18655728705582
  H   -2.52848465319179     -2.18139872697122       2.19503482387667
  H   -2.53789726330693     -0.92123070542459       0.91808314041791
  H   2.93450924351387     -0.24895254415583     -0.96651219630509
  H   1.34726781191814      0.54758485380511     -1.17667242968451
  H   1.66964282152211     -0.46091856149216      1.08420524394414
  H   1.84589310415772     -2.08125240106472      0.34648308687311
  H   -0.38911041499969     -1.08289189338230       2.22862987530074
  H   -0.15175933973042     -2.74228341192868       1.60279589222493
  K   -1.23664376152413     -2.01792154562549      -2.26738075435542
  O   -3.18636234952181     -0.19803958855603      -2.59721479166911
  H   2.24675329260510      0.72330020362976     -8.56805466660596
```

H	-1.59688437773346	1.81818349461623	-6.24009785041383
H	-3.61052084291707	0.21790431222602	-4.84845340522220
C	2.22181456238945	1.35829293319318	-7.67837978021689
H	0.20587382594540	0.76985392114857	-7.18215318902717
C	1.05967739560672	1.38357787248046	-6.89570973578222
C	3.33515887141592	2.12757656529389	-7.33796526171012
H	4.23623914805565	2.10055594708584	-7.95483662543922
C	-1.24681923695098	1.55898150065022	-5.22667170929472
C	-3.58915868528686	1.18462193686853	-4.35903094901639
H	-2.47673585746836	3.01236470094680	-4.20670877765985
C	1.01488102017095	2.19623752717455	-5.75220728109028
C	3.28250180754837	2.93530593527190	-6.19365472279770
C	-2.29664597119229	1.92658152863317	-4.19139457020650
O	-0.06074765509429	2.28908247551227	-4.92286986309719
Br	-4.58175041473288	2.23586500371771	-6.25139389475028
C	2.13451327006798	2.97304346856514	-5.40550014505601
H	-1.03628775685994	0.47502921133323	-5.19669357092330
H	-4.43306307741237	1.44838533483847	-3.73444656541416
H	4.14554852287509	3.54467052882143	-5.91251517866426
H	2.08210931269365	3.59954452548838	-4.51221867289655
H	-1.92166865199467	1.67213726633029	-3.18775602231825
H	-3.88453998187411	-0.86606976161948	-2.65088145661949
O	-3.49872843530208	1.15883521817098	-0.41530967585571
H	-3.37753249424321	0.61354113484892	-1.27711142561891
H	-4.20794975470547	1.78244130479983	-0.61420566365302

TS4-18C6

67
Coordinates from ORCA-job geom

C	-5.36164666292084	-2.93456646550005	-2.84501981977337
O	-4.12153899883912	-3.46583737286212	-3.27278395663900
C	-5.33480580271093	-2.70440897373820	-1.35289652959982
C	-4.09783520686388	-3.81853859864697	-4.64426308130353
C	-2.74671598792849	-4.38843726074553	-5.00795588374441
O	-1.75089946166302	-3.39697186907113	-4.85018595850519
O	-4.37155130447328	-1.71918294653159	-1.02763372479259
C	-0.45931960341180	-3.80969921207855	-5.25736573829553
C	0.54941271219702	-2.73517755675906	-4.92535518235013
C	-4.30217018675916	-1.44173345901016	0.35875967629540
C	-3.20506106968759	-0.44090803701509	0.63164868835447
O	-1.95001261697126	-1.01638239181406	0.32028627023671
O	0.68865503659866	-2.63011771073133	-3.51937416478919
C	-0.84496939275986	-0.19818489800615	0.65752871636119
C	1.65163621414517	-1.67352166546991	-3.11534189053200
C	1.70216996631996	-1.60392670437427	-1.60644623380178
C	0.43884419917363	-0.90211915773888	0.28416412725304
O	0.49263096403535	-1.05589298790284	-1.11927236376800
H	-6.18365382603351	-3.63883204851358	-3.07681343501955
H	-5.57558896245903	-1.98561417155120	-3.37491810891436
H	-6.34221723370990	-2.38049306711495	-1.02711390090384
H	-5.09928803079197	-3.65168468909367	-0.83054533096729
H	-4.31414831484775	-2.93141702335373	-5.27048103238838
H	-4.87383381271386	-4.57846322571680	-4.85895508761760
H	-2.78406007992667	-4.73710199028122	-6.05835331127077
H	-2.51820543548561	-5.26448058367884	-4.37002057166365
H	-0.44304490714451	-3.99382295367233	-6.34911057824167

```
  H   -0.18222654883613     -4.75392813703485     -4.75008928082664
  H   1.51893470689498     -3.00375769438989     -5.38808070259887
  H   0.22255832591059     -1.76562700691928     -5.34648587132163
  H   -4.10562473279608     -2.37364114102919      0.92296500964282
  H   -5.26395365227568     -1.02584876517581      0.71654912153222
  H   -3.24511216415526     -0.15679914325478      1.70080836283402
  H   -3.36576256675045      0.47698775682165      0.03285202353335
  H   -0.83860921332485      0.01193538102278      1.74454383128506
  H   -0.90951528015374      0.77064511074103      0.12517285425093
  H   2.65324008283816     -1.95458316603006     -3.49513528140361
  H   1.39166651168408     -0.67971417202442     -3.52622835815598
  H   2.55876480159124     -0.96913531284125     -1.30741125901830
  H   1.86600489630722     -2.61411251972257     -1.18328046866117
  H   1.29325364432776     -0.29472435571900      0.64087537218893
  H   0.49286204460892     -1.88905720777583      0.78354518552669
  K   -1.88920868127697     -1.68617048601638     -2.54377301598432
  O   -1.60847694392472      0.50150543525104     -3.98518110380258
  H   -2.19980284302636     -3.55588612174375    -10.28757834446326
  H   -3.96705683032589     -0.02967158470019     -7.75770683670779
  H   -4.86503375222306     -0.03788933576633     -4.80205910006327
  C   -1.64920737165079     -2.65780129283256     -9.99442401768199
  H   -3.13237568947044     -2.09513503725787     -8.53185041749291
  C   -2.18275096633239     -1.83126996762189     -8.99653242976551
  C   -0.43690815319738     -2.34941011496336    -10.61276671984041
  H   -0.03013232537249     -3.00017556588355    -11.39020731827942
  C   -3.16086819660967     -0.04119197910122     -6.99810017496478
  C   -4.60535672703357      0.94672083410380     -5.20425405418827
  H   -3.17317300164146      2.03445749846785     -6.35432945321425
  C   -1.48684715744651     -0.67395659093645     -8.60990390553934
  C   0.25127015319037     -1.19171542232782    -10.22365437889498
  C   -3.36577462076206      1.02874080329460     -5.94667229087516
  O   -1.91229648720734      0.18972461109325     -7.65241933568856
  Br  -6.46224509977382      1.23856241367789     -6.39923675467899
  C   -0.26480576521257     -0.35975704655182     -9.23307482089591
  H   -3.15507989182711     -1.04305561468233     -6.52913675415035
  H   -4.78161485917930      1.74752116146811     -4.48203748737675
  H   1.20160327540883     -0.93362434713869    -10.69884342088673
  H   0.26346895900107      0.54466356526825     -8.92279453714732
  H   -2.52186129151275      0.83399444695612     -5.01233414309065
  H   -1.31953878283154      1.32606713224734     -3.57218131675255
```

TS4-18C6-H2O

```
70
Coordinates from ORCA-job geom
  C   -5.52581860089756     -2.74961308442175     -2.61190335707409
  O   -4.37176620665453     -3.36537349010165     -3.15376329074974
  C   -5.32972077000971     -2.51326513924855     -1.13321301911364
  C   -4.50570321399718     -3.74667017452748     -4.51007398545957
  C   -3.26332026255566     -4.48154013295970     -4.95282497426590
  O   -2.14808530537213     -3.61128599373072     -4.90806381407657
  O   -4.27437813698416     -1.59358509026094     -0.92863695799154
  C   -0.93826651606250     -4.22704608322775     -5.31326027999026
  C   0.21004973928077     -3.25684840362357     -5.17059857152751
  C   -4.03780027604051     -1.29673014844122      0.43481034599176
  C   -2.87469689442555     -0.34115068032443      0.55812494995456
  O   -1.68814255834831     -0.97659731986122      0.11936888336742
```

O	0.41643379911840	-2.96088744791847	-3.80175395794308
C	-0.52078220281448	-0.19390035352762	0.29173799080813
C	1.52678959120814	-2.10662155576241	-3.56524995628216
C	1.71426076287735	-1.92538510430506	-2.07756945452883
C	0.68778465070681	-0.98412581442192	-0.15258573883866
O	0.60481794357598	-1.22848079634595	-1.54229909888511
H	-6.41039320802599	-3.40044263644078	-2.75040637502118
H	-5.72862346313200	-1.79118457537529	-3.12835171965296
H	-6.27312095385838	-2.11858621894104	-0.70861796664806
H	-5.10412801654882	-3.47059408085569	-0.62483603240847
H	-4.66886715753317	-2.85588835819407	-5.14699416213851
H	-5.37867868168774	-4.41505921241949	-4.63811847904648
H	-3.41905183418653	-4.85790368724120	-5.98230873130916
H	-3.08936814573354	-5.35547092040605	-4.29582773045250
H	-1.00663259608560	-4.54777857527374	-6.37068669884814
H	-0.74710948904426	-5.12701098411247	-4.69750180625060
H	1.11719042452211	-3.72040359471418	-5.60459671793564
H	0.00134732809837	-2.33057883350244	-5.73883988297684
H	-3.82111739217090	-2.22659980770762	0.99520969792719
H	-4.93353354954157	-0.82961111983478	0.88824576579646
H	-2.77912571953024	-0.03621409880742	1.61807583356546
H	-3.06545915074398	0.57101205121348	-0.04019864090422
H	-0.39651433183610	0.08284761445977	1.35644160468146
H	-0.59763837355325	0.74126220494595	-0.29609134393786
H	2.44747401701450	-2.55564484990895	-3.98547831465237
H	1.35535787258456	-1.12746451004866	-4.04995054351210
H	2.64673166958876	-1.35404383922614	-1.90332150403854
H	1.82275868842696	-2.90935854082469	-1.58109404614511
H	1.60009746156548	-0.40305442670844	0.08459382740792
H	0.74146368515382	-1.94017882129606	0.40358418331635
K	-1.96926543602571	-1.74856504601162	-2.67398520223135
O	-2.30282702073028	0.70285146226254	-3.71676841424389
H	-1.05404812008006	-3.28376199546067	-10.17927315613137
H	-3.51682893064950	-0.07060963807111	-7.97004828717992
H	-5.22438923572950	-0.20946153723381	-5.38339938372022
C	-0.64393034846066	-2.38499709005863	-9.71098119656995
H	-2.43297757016169	-2.03947765342169	-8.55598467435085
C	-1.43175826260130	-1.68011280671063	-8.79167100401780
C	0.64477947433973	-1.95624721587236	-10.03160958700172
H	1.25085612560972	-2.51353155559405	-10.74947375081491
C	-2.97344369297027	-0.06931309596116	-7.00638304465021
C	-4.91397784163674	0.79312417237497	-5.69159220411791
H	-3.30666650657302	1.99602591491793	-6.40089499758719
C	-0.91664290921514	-0.52445869015557	-8.18507253841187
C	1.15121582919119	-0.80082070481417	-9.42214796067853
C	-3.54259217951607	0.97297563094888	-6.06780228003698
O	-1.59504766221918	0.22364766512479	-7.26729284041060
Br	-6.42224621098008	0.93064668446087	-7.42154738827975
C	0.37943895569306	-0.08606485332705	-8.50845975979773
H	-3.06039421255815	-1.08158662535564	-6.57008363853264
H	-5.36840768513319	1.58570861306718	-5.09312096784597
H	2.15732216441551	-0.44836279478655	-9.66425535404637
H	0.76431991382439	0.82138100964646	-8.03725801851112
H	-2.96915428085410	0.85300068764527	-4.88857652268384
O	0.07987781168727	0.84434339896889	-4.87835028105837
H	-0.30116111765540	0.69181557065599	-5.75756391190571
H	-0.74497138969400	0.83904283909553	-4.31120837819097
H	-2.61687428766378	1.42611428789640	-3.15779118720205

5. References

1. Ávila, E. P.; de Almeida, M. V.; Valle, M. S.; Pliego, J. R., *ACS Org. Inorg. Au* **2025,** *5*, 69-83
